# Compartmentalized gene regulatory network of the pathogenic fungus *Fusarium graminearum*

**DOI:** 10.1111/nph.13912

**Published:** 2016-03-14

**Authors:** Li Guo, Guoyi Zhao, Jin‐Rong Xu, H. Corby Kistler, Lixin Gao, Li‐Jun Ma

**Affiliations:** ^1^ Department of Biochemistry and Molecular BiologyUniversity of Massachusetts AmherstAmherstMA01003USA; ^2^ Department of Electrical & Computer EngineeringUniversity of Massachusetts AmherstAmherstMA01003USA; ^3^ Department of Botany and Plant PathologyPurdue UniversityWest LafayetteIN47907USA; ^4^ USDA‐ARSCereal Disease LaboratoryUniversity of MinnesotaSt PaulMN55108USA

**Keywords:** Bayesian network inference, cell circuits, *Fusarium graminearum*, modularity, network rewire and fungal pathogenesis

## Abstract

- Head blight caused by *Fusarium graminearum* threatens world‐wide wheat production, resulting in both yield loss and mycotoxin contamination.
- We reconstructed the global *F. graminearum* gene regulatory network (GRN) from a large collection of transcriptomic data using Bayesian network inference, a machine‐learning algorithm. This GRN reveals connectivity between key regulators and their target genes. Focusing on key regulators, this network contains eight distinct but interwoven modules. Enriched for unique functions, such as cell cycle, DNA replication, transcription, translation and stress responses, each module exhibits distinct expression profiles.
- Evolutionarily, the *F. graminearum* genome can be divided into core regions shared with closely related species and variable regions harboring genes that are unique to *F. graminearum* and perform species‐specific functions. Interestingly, the inferred top regulators regulate genes that are significantly enriched from the same genomic regions (*P *< 0.05), revealing a compartmentalized network structure that may reflect network rewiring related to specific adaptation of this plant pathogen.
- This first‐ever reconstructed filamentous fungal GRN primes our understanding of pathogenicity at the systems biology level and provides enticing prospects for novel disease control strategies involving the targeting of master regulators in pathogens. The program can be used to construct GRNs of other plant pathogens.

## Introduction

Fusarium head blight (FHB), caused by the filamentous ascomycete *Fusarium graminearum* (*F. graminearum*), is one of the most devastating diseases in wheat (*Triticum aestivum*), barley (*Hordeum vulgare*) and other small grains around the world (Goswami & Kistler, 2004; Leslie & Summerell, 2006; Rep & Kistler, 2010; Guo & Ma, 2014). FHB results in yield loss and contamination of grains with mycotoxins (Leslie & Summerell, 2006), fungal secondary metabolites toxic to animals, including humans. The management of FHB remains challenging because of a lack of effective resistant wheat cultivars and the prevalence of pathogen resistance to fungicides. The understanding of *F. graminearum* pathobiology at the systems level is vital to effective disease and mycotoxin management.

From a systems biology perspective, any biological process, such as growth, reproduction or host invasion, is accomplished by genome‐encoded molecular components that are orchestrated and assembled into interconnected cell circuits (Kim *et al*., 2009). A gene regulatory network (GRN) depicts the relationships between regulatory components (e.g. kinases and transcription factors (TFs)) and their target genes (e.g. enzymes and structural proteins), key components of cell circuits. Among fungal species, GRN studies have extensively focused on yeasts, such as *Saccharomyces cerevisiae* (Pe'er *et al*., 2001, 2002; Guelzim *et al*., 2002; Lee *et al*., 2002; Segal *et al*., 2003; Nachman *et al*., 2004; Kim *et al*., 2006; Hu *et al*., 2007; Darabos *et al*., 2011) and *Candida albicans* (Homann *et al*., 2009; Ramachandra *et al*., 2014). By contrast, the reconstruction of GRN for filamentous fungi remains in its infancy. The best understood *F. graminearum* transcriptional regulation system is the *Tri* gene cluster, which is modulated by two internal TFs, Tri6 and Tri10 (Seong *et al*., 2009; Nasmith *et al*., 2011), and directs the biosynthesis of trichothecene mycotoxins (Goswami & Kistler, 2004; Rep & Kistler, 2010). A chromatin immunoprecipitation sequencing (ChIP‐seq) analysis (Nasmith *et al*., 2011) revealed that Tri6 physically binds to the promoter sequences of six *Tri* genes and 192 non‐*Tri* genes by recognizing the ‘TNAGGCC’ motif (Nasmith *et al*., 2011). It remains to be determined how cellular components work systemically to regulate *F. graminearum* development, invasive growth and virulence.

There are rich resources to facilitate such an endeavor. The *F. graminearum* genome has been fully sequenced (Cuomo *et al*., 2007) and putative regulatory proteins have been comprehensively annotated (Guldener *et al*., 2006a; Park *et al*., 2008, 2011; Wong *et al*., 2011). In addition to the *Tri* cluster, over 100 pathogenicity‐related genes have been characterized, including signal proteins (SPs), such as G proteins (Yu *et al*., 2008), small GTPases (Bluhm *et al*., 2007) and mitogen‐activated protein kinases (MAPKs) (Urban *et al*., 2003; Jenczmionka & Schafer, 2005). Genome‐wide functional studies of *F. graminearum* TFs (Son *et al*., 2011) and protein kinases (PKs) (Li *et al*., 2011) have linked 170 TFs and 64 PKs to fungal growth, development and virulence. An *F. graminearum* protein–protein interactome database (FPPI) has been constructed (Zhao *et al*., 2009). Furthermore, over 100 transcriptome datasets, covering diverse biological states, such as plant infection (Boddu *et al*., 2006; Guldener *et al*., 2006b; Lysoe *et al*., 2011), sexual development (Qi *et al*., 2006; Hallen *et al*., 2007; Lee *et al*., 2010; Sikhakolli *et al*., 2012), conidial germination (Seong *et al*., 2008) and mycotoxin production (Gardiner *et al*., 2009; Seong *et al*., 2009; Jonkers *et al*., 2012), are available in the PLEXdb public database (Dash *et al*., 2012) (http://www.plexdb.org). Large collections of expression profiles, either generated in our research or obtained from PLEXdb, established a foundation for the systemic inference of the *F. graminearum* GRN.

Many sophisticated computational programs, including Gaussian graphical models (Kishino & Waddell, 2000; Schafer & Strimmer, 2005a,b), probabilistic Boolean networks (Kauffman, 1969; Glass & Kauffman, 1973; Shmulevich *et al*., 2002) and Bayesian networks (BNs) (Friedman *et al*., 2000; Pe'er *et al*., 2002; Segal *et al*., 2003), have been developed for GRN reconstruction. This study adopted MinReg (Pe'er *et al*., 2002), a scalable BN model that defines regulatory relationships using expression data, and has been successfully used to reconstruct yeast (Pe'er *et al*., 2002) and mammalian (Amit *et al*., 2009) GRNs. A probabilistic graphical model, BN combines multivariate probability distributions and captures the properties of conditional independence between variables. Because of its ability to describe complex stochastic processes and to provide clear methodologies for learning from noisy observations, BN has emerged as a common and attractive model for the reconstruction of GRNs from expression data (Friedman *et al*., 2000; Vignes *et al*., 2011).

Here, we report the first genome‐wide *F. graminearum* GRN by harnessing available genomic, transcriptomic and functional data. Remarkably, the predicted regulators were functionally correlated with target genes. Topologically, the GRN was divided into eight distinct functional modules that were differentially regulated under various biological conditions, in agreement with the finding that each module performed distinct functions. Finally, we provide evidence that the *F. graminearum* regulatory network is compartmentalized in the core vs species‐specific genomic regions, providing insight into the circuit rewiring that may contribute to fungal genome evolution and specialization. Key components of the GRN are promising candidates for devising novel strategies to control this destructive disease. The computational program used here is also suitable for network inference in a broad spectrum of phytopathogenic fungi.

## Materials and Methods

### Strains

*Fusarium graminearum* Schwabe PH1 wild‐type and mutants (Supporting Information Table S1) were kindly provided by Dr Jin‐Rong Xu at Purdue University, and have been described previously (Wang *et al*., 2011).

### RNA preparation, chip hybridization and analysis

For the 27 sets of microarray data generated in this study (Table S1), total RNAs were isolated from fungal hyphae harvested from 36‐h‐old complete medium cultures with TRIzol^®^ Reagent (Invitrogen, Carlsbad, CA, USA), following the manufacturer's recommendations. Microarray hybridization was performed using the Affymetrix Fungal Multigenome ExonChip, and data processing and analysis were conducted as described previously (Guo *et al*., 2016). Three biological replicates were conducted for each experiment. For data quality control, Pearson correlation coefficients (*r*) were calculated, and scatterplots were created for each possible pair of biological replicates (Fig. S1) using the R package ggally.

### Combined data and normalization

One‐hundred and sixty‐six *F. graminearum* microarray samples, subjected to 55 experimental conditions (Table S1), were obtained from PLEXdb. As these datasets were generated from different sources, expression levels in all samples were calculated uniformly from raw microarray data, as described previously (Guo *et al*., 2016). The expression of each gene under each experimental condition was classified into three discrete statuses: normal, upregulated and downregulated (Table S2). The normalized data were used as input in our network inference program.

### MinReg algorithm implementation

The MinReg algorithm, based on the BN model (Pe'er *et al*., 2006), was adapted for our GRN inference. BN learning includes two aspects: learning the parameters of the conditional probability distribution for genes as nodes and learning the BN structure. With thousands of gene nodes in a regulatory network, it is an NP (nondeterministic polynomial time)‐complete problem to enumerate all possible network structures and optimize the parameters. Under the assumption that only a certain number of regulators regulate a target gene, the MinReg algorithm uses a heuristic greedy strategy to add regulators, one by one, by selecting the regulator that can increase the BN score (BNS) the most. If adding a new regulator does not significantly increase the overall BNS, the algorithm terminates. In our implementation, we selected the candidate regulator with the highest BNS as a key regulator in each iteration. The number of key regulators was finalized based on the score distribution of BNS increase. Target genes were assigned to respective regulators under the constraint of a limited number of parents, which is denoted as *d*. For the first set of *d* regulators (parents for all target genes), all possible combinations of *d* regulators from the candidates were enumerated to select the combination that gave the highest BNS. Then, for a new regulator to be added, the parents of each target gene were combined to select the combinations of *d* regulators that gave the highest BNS. After summing all the BNSs, the candidate regulator that gave the highest score increase was added to the network. When selecting the (*k *+ 1)^th^ regulator, if the slope of the score increase was less than the threshold, the program stopped and selected the *k* regulators as the final parents of the target genes. The source codes and detailed instructions for searching the *F. graminearum* GRN can be accessed at: http://rio.ecs.umass.edu/Fusarium_GRN.

### *R*_GO_ score calculation

If regulators were SPs, functional enrichment using FunCat (Ruepp *et al*., 2004) was performed on target genes of each SP regulator to obtain significantly enriched terms with a *P* value threshold of 0.05. The significantly enriched terms were compared with functional terms of the SP regulator. The functional capture rate *R*_GO_ was then calculated by dividing the number of shared functional terms by the total number of regulator functional terms.

### Permutation test

A permutation test was performed to compare the *R*_GO_ scores of our inferred regulatory network and the randomly generated regulator–target gene relationship. In each permutation, 120 regulators were randomly selected from candidate regulators. Each randomly selected regulator was mapped to the *F. graminearum* network with a set of randomly selected target genes of the same size. With the new random target genes, the *R*_GO_ score for each random regulator was calculated following a FunCat analysis. After running permutation tests 100 times, the average distribution of the *R*_GO_ score was compared with that of the inferred network.

### Function prediction of TFs

Predicted TF regulators were evaluated using two different pipelines. First, the 75 *F. graminearum* TFs were searched for homologs in the *S. cerevisiae* genome (http://www.yeastgenome.org/) using Blastp (e < 1e‐10). For the identified *F. graminearum* TF homologs, FunCat analysis was performed for target genes and enriched functions were compared with *S. cerevisiae* TF homolog functions to identify consensus functions. Second, TF binding motif enrichment was performed for target genes of each *F. graminearum* TF homolog using degenerate TF binding motifs predicted previously (Kumar *et al*., 2010). The enriched TF binding motif was then compared with the binding motif in the *S. cerevisiae* TF homolog, if present in the YeTFaSCo database (http://yetfasco.ccbr.utoronto.ca), to identify similar binding motifs based on matching score computation.

### TF binding site matching score

Generally, the TF binding motif is represented as a position weight matrix (PWM) or sequence logo. The motif matching score function was derived to measure the distance between the most enriched *F. graminearum k*‐mer motif and the known *S. cerevisiae* motif. The matching score for the most enriched motif *m*_*g*_ and the published *S. cerevisiae* motif *m*_*y*_ was calculated in Eqn 1:

(Eqn 1) $$m\text{‐score}=\max_{1\leq t\leq m-n}\left\{\frac{\sum_{i=1}^{n}M_{m_{g}\left[i\right],i+t}}{\sum_{i=1}^{n}\max_{s\in \Sigma }M_{s,i+t}}\right\}$$

The PWM for *S. cerevisiae* motif *m*_*y*_ is represented as a frequency matrix of *M*_*k,j*_, where *k* is one of possible nucleotides Σ = {A, C, G, T} and *j* is the position. The length of motif *m*_*y*_ is denoted as *m* and the length of motif *m*_*g*_ is denoted as *n*. The nucleotide at position *i* of *m*_*g*_ is denoted as *m*_*g*_[*i*]. When *m*_*g*_ is aligned with *m*_*y*_ at position *t* of *m*_*y*_, the total matching score equals the summation of frequency *M*_*k,j*_ at each aligned position. The denominator is the normalization factor to remove the influence of the maximum frequency. From all the possible aligned positions, the largest matching score is the *m*‐score.

### Modularity analysis

Regulatory modules in the *F. graminearum* GRN were identified using the modularity function in Gephi (Bastian *et al*., 2009) employing randomization and a resolution score of 1.0. Genes of each regulatory module were exported and functional enrichment was conducted using FunCat. Gene expression levels of each module were extracted from master data tables using inhouse Python scripts, clustered and visualized using MEV (http://www.tm4.org/mev.html).

### GRN–FPPI overlapping network analysis

To identify overlapping elements between the FPPI network and the GRN inferred in this study, genes and their interactions present in both networks were captured using inhouse Python scripts. FPPI network data were kindly provided by Professor Weihua Tang at the Institute of Plant Physiology and Ecology of the Chinese Academy of Sciences.

### Statistical analysis

Statistical analyses, including Student's *t*‐test and Fisher's exact test, were conducted using the *stats* functions in the R programming language (http://www.r-project.org). The *F. graminearum* genome has been divided previously into core and species‐specific (FS) compartments containing *c*. 9700 and 3600 genes, respectively (Ma *et al*., 2010). For the top‐ranked 20 regulators for the two compartments, we performed Fisher's exact test using the *fisher.test* function in R. Our data provide strong support for the biased distribution of top regulators for both regions. Core regulators (19/20) are significantly enriched in the core genome compared with random chance (9700/13 300) (*P *= 0.02281), whereas FS regulators (1/20) are under‐represented in the core genome. Similarly, FS regulators (12/20) are significantly enriched in the FS genome compared with random chance (3600/13 300) (*P *= 0.002), whereas core regulators are significantly under‐represented in the FS genome.

### Data access

Microarray data generated in this work using the Affymetrix Fungal Multigenome ExonChip (Guo *et al*., 2016) are available at the Filamentous Fungi Gene Expression Database (Zhang & Townsend, 2010) (Experiment ID: 241, 242, 246 and 247) (http://bioinfo.townsend.yale.edu/). The GRN inferred in this work, as well as related datasets, source codes and instructions for using the network, can be accessed at: http://rio.ecs.umass.edu/Fusarium_GRN.

## Results

### Global *F. graminearum* GRN inference

**Figure 1:**
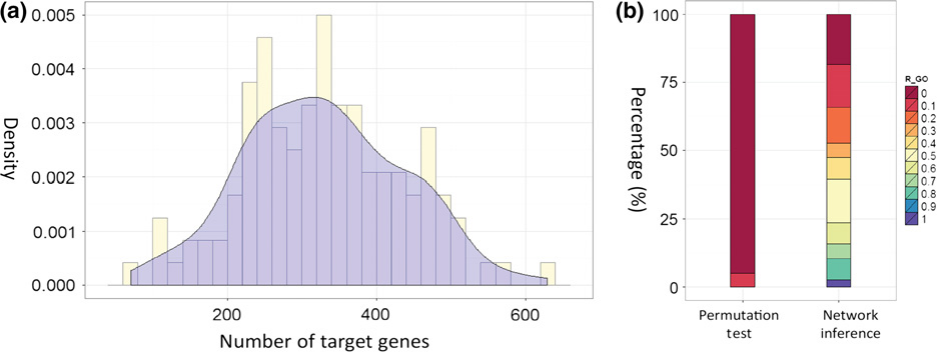
Summary of the *Fusarium graminearum* gene regulatory network (GRN). (a) The abundance of target genes for all regulators is summarized in a histogram and a density curve. *x*‐axis, number of target genes; *y*‐axis, density of regulators. (b) A stack bar plot comparing the *R*_GO_ (function capture rate) distribution for signal protein (SP) regulators in the inferred GRN and the random network obtained by a permutation test (see the Materials and Methods section). *R*_GO_ equals the ratio of functional terms shared by a regulator and its target genes (significantly enriched: *P *< 0.05) to the total functional terms of the regulator (see the Materials and Methods section). Color scale: *R*_GO_ ranges from 0 to 1.

In this study, we inferred the *F. graminearum* GRN by adapting MinReg, a machine‐learning algorithm based on a BN model (Pe'er *et al*., 2002) which treats the expression level of each gene as a random variable and attempts to estimate the structural features of the dependences in their joint probability distribution from the data. To collect sufficient expression data for reliable network prediction, we generated 27 expression profiles based on nine experiments (see the Materials and Methods section) (Table S1) and acquired 166 transcriptomic datasets curated at the Plant Expression database (PLEXdb) (Dash *et al*., 2012). Expression data from both sources were combined and normalized, generating a global gene expression data matrix as input data (Table S2). A total of 968 candidate regulator genes, including 660 putative TFs and 308 SPs (Table S3), were identified based on current functional annotations and published studies (Cuomo *et al*., 2007; Wong *et al*., 2011). Overall, the global BNS increased when a new regulator was added (Fig. S2). At the beginning of the BNS distribution curve, there was a linear correlation between the global BNS value and the increase in regulators. The increase declined significantly after 120 regulators had been added. Theoretically, each candidate regulator can be fitted into the GRN; however, doing so is computationally costly and creates statistical noise which probably leads to spurious dependences with a relatively small sample size. Therefore, we terminated our search with a parsimonious set of major regulators and limited our search to a simple network structure in an effort to balance the high resolution of the learned networks with statistical robustness (see the Materials and Methods section). We defined these 120 regulators, which can be accessed at the website http://rio.ecs.umass.edu/Fusarium_GRN, as top regulators of the *F. graminearum* GRN (Fig. S3). The inferred top regulators included 75 TFs and 45 SPs. The number of target genes for each regulator ranged from 71 to 629, with an average of 329 (Fig. 1a). Because the expression data covered diverse biological states with an emphasis on pathogenesis, we aimed to deliver a reduced network structure that described the regulation of fundamental biological processes with an emphasis on the regulation of fungal pathogenesis. This framework can be further fitted with expression data under different perturbations to understand the regulation of specific biological functions.

### Validation of the predicted *F. graminearum* GRN

#### Top SP regulators share functions with their target genes

Regulators and their target genes are usually involved in the same biological processes (Pe'er *et al*., 2006). This is strongly supported by the top SP regulators identified in our study and the enriched gene ontology (GO) terms of their target genes. For example, 38 of the 45 inferred top SP regulators have been functionally annotated, which enables a comparison of the GO terms assigned to each regulator and its target genes using the ratio score function *R*_GO_ (see the Materials and Methods section). The scores range from 0 to 1. A score of 1 reflects complete functional overlap, as all GO terms assigned to a regulator are enriched in the GO terms assigned to its target genes. By contrast, a score of 0 indicates a lack of functional overlap. Of the 38 annotated regulators, the average *R*_GO_ score was 0.34, significantly higher than the randomized permutation test average *R*_GO_ score of 0.0051 (Student's *t*‐test, *P *= 0.00205). Specifically, the *R*_GO_ score for the regulator RAS2 (FGSG_10114) was 1, and six regulators had *R*_GO_ scores of ≥ 0.7, and about half of the SP regulators (18) had *R*_GO_ scores that were ≥ 0.4 (Fig. 1b; Table S4).

These top SP regulators, which act as controlling centers of the GRN and regulate many key processes essential for survival and proliferation, were found to be mostly involved in housekeeping cell functions, such as metabolism, cell cycle control, cellular transport, transcription, translation and cell growth (Table S4). For example, RAS2 (FGSG_10114) was predicted to regulate 557 target genes in the *F. graminearum* GRN. Empirically, the *ras2* mutant showed multiple defects, including defects in vegetative growth, mating and virulence (Bluhm *et al*., 2007), even though the mutant was viable. Interestingly, these 557 target genes included 38 genes involved in the cell cycle, 41 in stress responses and 34 in cell polarity and ascospore development. Among the 45 SP regulators, at least three, that is, FGSG_00677 (subunit of casein kinase 2, CK2), FGSG_01137 (*FgMPS1*) and FGSG_09660 (*FgPKC1*), are essential (Fig. S4), and no viable knockout mutants could be experimentally obtained for these (Wang *et al*., 2011). Mutants of the other eight regulators showed phenotypic defects in one or more processes, including vegetative growth, sporulation, toxin production and virulence (Wang *et al*., 2011), supporting their involvement in essential cellular functions as top regulators (Fig. S4).

#### Most predicted top TF regulators are functionally conserved

**Figure 2:**
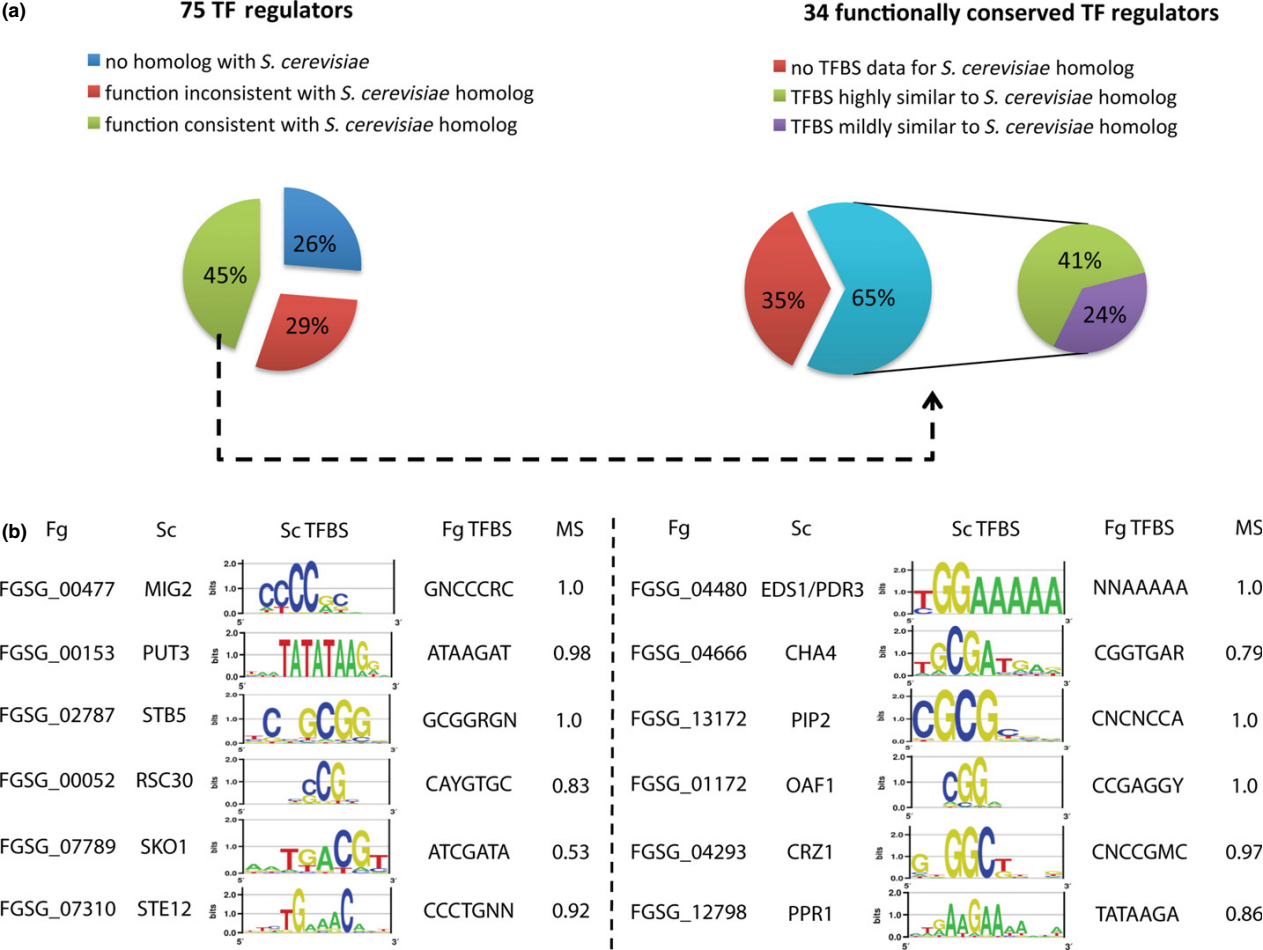
Transcription factor (TF) regulators inferred in the *Fusarium graminearum* gene regulatory network (GRN) have exceptional functional or TF binding site (TFBS) consistency with *Saccharomyces cerevisiae* homologs. (a) Pie charts summarizing the validation of 75 TF regulators inferred in the *F. graminearum* GRN compared with *S. cerevisiae* homologs in terms of function and TFBS. Dotted lines and arrows represent a further breakdown of functionally conserved TFs based on TFBS knowledge. (b) Summary of functionally conserved *F. graminearum* and *S. cerevisiae* TFs that share highly similar TFBSs, suggested by motif MSs (matching scores). MS computation details are provided in the Materials and Methods section.

Unlike SPs, most TFs in the *F. graminearum* genome have limited GO annotations associated with biological processes and lack functional annotations other than nucleic acid binding. Validation methods based on such incomplete information could amplify both false‐positive and false‐negative signals. Therefore, we took a homologous‐based approach, comparing the predicted top regulators with homologs in the best‐characterized model fungal genome, *S. cerevisiae*. Among the 75 predicted top TF regulators, 56 had putative *S. cerevisiae* homologs (e < 1e‐10). Transferring the functional annotation of yeast proteins to their *F. graminearum* homologs enabled us to perform an annotation‐based validation similar to that described for SPs. Interestingly, the functions of 34 of the 56 top TFs, as suggested by their yeast homologs, were confirmed by the enriched functional categories inferred by their target genes (see the Materials and Methods section) (Fig. 2a; Table 1). These 34 conserved regulators regulate a wide range of cellular processes, including the cell cycle, RNA processing during transcription and translation, mitochondrial functions, mating, stress responses and chromatin dynamics (Table 1).

**Table 1:** Summary of conserved *Fusarium graminearum* (*Fg*) transcription factor (TF) regulators homologous to those in *Saccharomyces cerevisiae* (*Sc*)

*Fg* TF regulators	*Fg* degenerate TFBS	*Sc* homologs	*Sc* TFBS evidence	Consensus function annotation	Motif matching score
FGSG_04666	CGGTGAR	*CHA4*	No	Cell cycle	–a
FGSG_07097	CNCCAAN	*YOX1*	Yes	Cell cycle control	0.51
FGSG_07924	CGACNNC	*ZNF1*	No	Cell cycle, cell component biosynthesis, cell differentiation, signal transduction	–
FGSG_07310	CCCTGNN	*STE12*	Yes	Cell cycle, cytokinesis, mating, cell type differentiation, stress response	0.92
FGSG_09807	YGCGACN	*CEF1*	No	Cell cycle, RNA processing, mRNA splicing	–
FGSG_08349	NGTSACG	*UME6*	Yes	Cell cycle, transcription, cell transport	0.57
FGSG_00385	CCNCNTC	*NHP6B*	Yes	Chromatin remodeling, condensation	–
FGSG_08719	NGCCNCA	*MYO1*	No	Cytoplasmic and nuclear protein degradation, cell cycle, cytoskeleton structure	–
FGSG_07789	ATCGATA	*SKO1*	Yes	Disease, virulence, defense	0.53
FGSG_00477	GNCCCRC	*MIG2*	Yes	Fungal cell differentiation	1
FGSG_00318	CCCCGSA	*BAS1*	Yes	Metabolism, energy, transport	0.71
FGSG_00717	AAAANTT	*TIM10*	No	Mitochondria, mitochondria transport, heat shock stress response	–
FGSG_09892	GATGNCN	*MPE1*	No	mRNA synthesis, transcriptional control	–
FGSG_00153	ATAAGAT	*PUT3*	Yes	Nitrogen utilization	0.98
FGSG_04480	NNAAAAA	*EDS1*	Yes	Nonvesicular transport, stress response	1
FGSG_00800	CCNCCNC	*HAL9*	Yes	Osmotic and salt stress response, stress response	1
FGSG_13172	CNCNCCA	*PIP2*	Yes	Oxidation of fatty acids, cell cycle and DNA processing	1
FGSG_06516	GCKGACT	*STB5*	Yes	Oxygen and radical detoxification	1
FGSG_01172	CCGAGGY	*OAF1*	Yes	Peroxisome, fatty acid, mitochondria	1
FGSG_01411	NNCGCGT	*PFA4*	No	Protein modification, degradation, cell cycle regulation	–
FGSG_06220	GNGGGGY	*SUI2*	No	Protein synthesis, translation, translation initiation	–
FGSG_05012	AGCTNCN	*ARC1*	No	Protein synthesis, tRNA aminoacylation, tRNA binding, stress response	–
FGSG_00052	CAYGTGC	*RSC30*	Yes	Regulation of ribosomal protein genes	0.83
FGSG_04554	YNATTGG	*DPS1*	No	RNA modification, rRNA processing	–
FGSG_06168	RAAAAAN	*RSF2*	Yes	rRNA processing, protein synthesis, transcription	0.84
FGSG_11996	ACGTMAT	*ECM22*	Yes	Sterol metabolism	0.62
FGSG_00713	NCTCCCN	*STB5*	Yes	Stress response	0.61
FGSG_12798	TATAAGA	*PPR1*	Yes	Stress response	0.86
FGSG_02696	NACGTCA	*PPR1*	Yes	Stress response	0.84
FGSG_02787	GCGGRGN	*STB5*	Yes	Stress response	1
FGSG_04293	CNCCGMC	*CRZ1*	Yes	Stress response, calcium binding	0.97
FGSG_01638	GCGNCAN	*HAP1*	Yes	Transcription regulation	0.64
FGSG_00324	CNCCCNC	*SNT1*	No	Transcription, chromatin regulation, meiosis	–
FGSG_05498	GANGCGN	*BUR6*	No	Transcription, chromosomal cycle	–

TFBS, transcription factor binding site.

a Score not calculated.

A comprehensive mutagenesis study generated and characterized all 660 TFs in the *F. graminearum* genome, showing that 170 TF deletion mutants have at least one phenotypic defect (Son *et al*., 2011), including 13 of the 75 predicted top TF regulators (Fig. S4). Interestingly, 10 of these 13 TFs were found to be conserved in *S. cerevisiae*. For example, FGSG_07310 was found to be the homolog of *S. cerevisiae STE12,* which is activated by the MAPK cascade in response to pheromones and controls mating (Elion *et al*., 1993). The FGSG_07310 deletion mutant is unable to reproduce sexually, confirming the essential role of FGSG_07310 in mating (Son *et al*., 2011). Deletion mutants of four of these TFs, FGSG_00324, FGSG_00385, FGSG_00477 and FGSG_08719 (Fig. S4), had multiple defects (Son *et al*., 2011), consistent with the fundamental biological processes they share with their *S. cerevisiae* homologs, such as transcription, and chromatin and cytoskeleton structure (Table 1).

TFs control the transcription of their target genes by binding to their respective regulatory sequences, called TFBSs, at the promoter regions of the target genes. It has been reported repeatedly that the sequence of a TFBS can be preserved in a conserved TF over evolutionary distance, for instance from unicellular yeasts to filamentous fungi (Kumar *et al*., 2010; Wittkopp & Kalay, 2012). Based on the set of computationally predicted TF *cis* elements in the *F. graminearum* genome (Kumar *et al*., 2010), we identified the most enriched TF binding sites (Student's *t*‐test) among target genes for each inferred top TF regulator (Table S5). On average, about one‐quarter of the predicted target genes of each TF shared an enriched binding site. We further developed a score function based on PWM to measure the sequence similarity between the enriched *F. graminearum* binding motifs and the corresponding ones predicted in the Yeast Transcription Factor Specificity Compendium (see the Materials and Methods section). The score ranges from 0 to 1, representing the least to the most matches. Among the 34 functionally conserved *F. graminearum–S. cerevisiae* TFs, 22 (65%) shared TF binding sequences with *S. cerevisiae* homologs (Fig. 2a; Table 1). The conserved binding motifs included the TFBS for *MIG2*,* PUT3*,* EDS1*,* STE12* and *STB5,* with matching scores of higher than 0.8 (Fig. 2b).

In summary, validation of the reduced *F. graminearum* GRN confirms its reliability in depicting the relationship between top regulators, both SPs and TFs, and their target genes. The conservation between *F. graminearum* and *S. cerevisiae* for both the predicted top TFs and their TFBSs suggests that most predicted top regulators in this reduced network structure perform conserved housekeeping functions.

### Modularity is a key feature of the *F. graminearum* GRN

**Figure 3:**
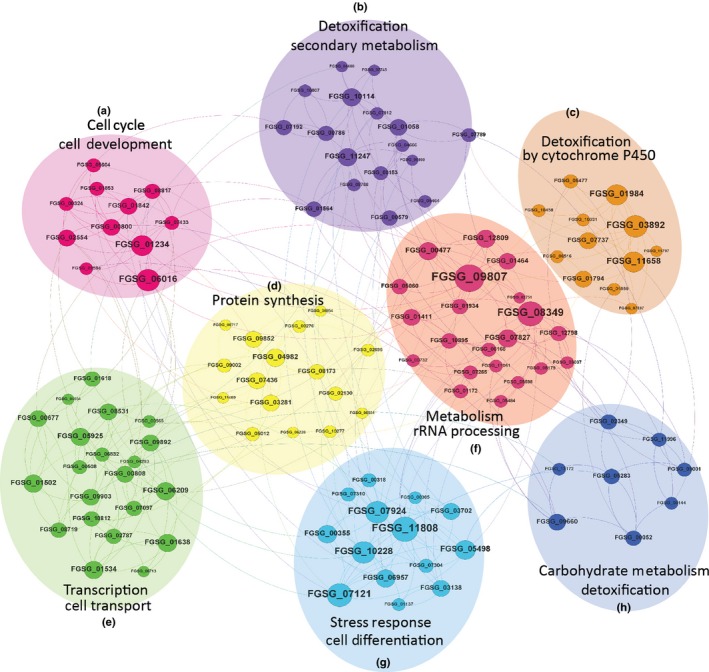
The *Fusarium graminearum* gene regulatory network (GRN) consists of eight regulatory modules that are regulated under different biological states. Visualization of the *F. graminearum* gene regulatory network, which is divided into eight modules (a–h). Only the regulator nodes are shown. Modules (shaded circles) and their enclosed regulator nodes are colored. The size and label of the nodes are proportional to the number of target genes for each regulator. Edges are nonweighted, simply showing inferred regulatory relationships. The functional annotation of each module represents the most significantly enriched gene ontology terms among target genes within the module.

**Figure 4:**
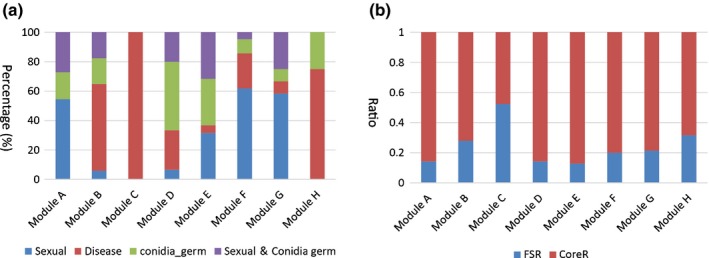
Dissection of functional association in regulatory modules of *Fusarium graminearum*. (a) Bar graphs summarizing the number of regulators belonging to the four major regulator clusters: Sexual, Disease, conidia_germ (conidia germination) and Sexual&Conidia germ, equivalent to the clusters shown in Fig. 3. The regulators were counted for each module and plotted. Bar colors represent the four clusters. (b) Core genome and *F. graminearum*‐specific distribution of regulators for each regulatory module. FSR, *F. graminearum*‐specific genome regulators; CoreR, core genome regulators.

The GRN usually contains interconnected functional modules that have specific functions in biological processes (Segal *et al*., 2003). Multiple software packages, such as Gephi (Bastian *et al*., 2009), were designed to extract tightly connected subnetworks from larger networks (Blondel *et al*., 2008), including social networks such as Facebook (Akhtar *et al*., 2013), as well as transcription regulatory networks (Sanz *et al*., 2011). Using Gephi, the *F. graminearum* GRN can be reproducibly divided into eight regulatory modules (Fig. 3). GO term enrichment tests (Table S6) confirmed that each module was functionally independent and was important for certain biological functions (Fig. 4). However, some functions were controlled by multiple modules (Tables 2, S6). For example, module A was found to function in the cell cycle, remodeling of chromatin structure and cytoskeletal organization, and cell polarity. Modules D, E and F were found to regulate translation, transcription and DNA replication, respectively. Module G was found to control cell differentiation and stress responses. Although modules B, C and H were all found to be involved in the regulation of cell detoxification processes, they were enriched for different cellular processes. For instance, module B was involved in secondary metabolism, including lipid, fatty acid and isoprenoid metabolism; module C was highly enriched for cytochrome P450‐related detoxification processes; and module H primarily regulated carbohydrate metabolism and detoxification.

**Table 2:** Summary of module statistics: the number of target genes and regulators in each module and the most significantly enriched functional terms in target genes ranked by *P* values (low to high)

Module	No. target genes	No. FS target genes (percentage)^a^	FS enrichment (*P* value)^b^	No. regulators	Significantly enriched biological processes^c^
A	2743	367 (13.4)	Under (5.4e‐38)	11	Cell cycle; cytoskeleton; budding; cell polarity; chromosomal structure; G‐protein‐mediated cell signaling
B	5066	1539 (30.4)	Over (1.1e‐3)	17	Detoxification; degradation of exogenous compounds; nonvesicular cellular import; secondary metabolism
C	3195	1658 (52)	Over (1.3e‐71)	12	Degradation of exogenous compounds; detoxification involving cytochrome P450; polysaccharide metabolism
D	3295	594 (18)	Under (3.4e‐18)	16	Protein synthesis; unfolded protein response; mitochondrion; stress response
E	4440	561 (12.6)	Under (3.8e‐63)	21	Transcription; cellular transport; cytoskeleton; cell cycle; mitochondrion; stress response
F	6807	1693 (24.8)	Under (8.4e‐3)	21	Metabolism; rRNA processing; Ori recognition and priming complex formation; vitamin/cofactor transport
G	4212	861 (20.4)	Under (6.5e‐12)	14	Stress response; fungal cell type differentiation; ascospore development; cell wall; G‐protein‐mediated signal transduction
H	3012	1151 (38.2)	Over (1.2e‐17)	8	Carbohydrate metabolism; detoxification by degradation; aminosaccharide catabolism

a FS, *Fusarium graminearum* specific. Percentage, FS target gene ratio per module.

b FS enrichment: enrichment test (two‐sided Fisher's exact test) for FS target genes over total target genes per module, compared with total FS genes (3600) against whole genome (13 300). Over, over‐representation or enrichment (*P* < 0.05). Under, under‐representation or lack of enrichment (*P *< 0.05).

c Significantly enriched biological processes using FunCat database (ranked by FunCat *P*‐value in ascending order). Complete FunCat results for all modules are available in Supporting Information Table S6.

Such functional affiliation was also supported by the findings that most regulators and target genes in modules B, C and H were upregulated during plant infection (Fig. S5) and that the gene expression profiles of modules B, C and H were distinct from those of the other modules (Fig. S5; Table 2). For instance, module A (cell cycle and cell development module) was preferentially expressed during sexual reproduction stages, but module D (translation module) was induced during conidial germination processes (Fig. S5). To quantify the functional affiliation of each module, we co‐clustered the expressed profiles of the 120 top regulators and the expression profiles used for the *F. graminearum* GRN inference employing a hierarchical clustering (HCL) algorithm (Fig. S6), and observed distinct clustering patterns for the regulatory modules (Fig. 4a), in agreement with their functional annotations. The most significant affiliation was in module C, where all regulators were found to be related to the disease phenotype (Fig. 4a) and were strongly induced during plant infection (Fig. S5). In addition to module C, modules B (57% of regulators) and H (70%) were also substantially correlated with disease phenotypes. Of 22 candidate FS effectors reported previously (Brown *et al*., 2012), 12, 12 and eight were found in modules B, C and H, respectively. Although all modules contain effector genes, B and C have the highest ratio of effector genes per module (Table S7). By contrast, modules A (80%), F (68%) and G (85%) were major contributors to sexual development. Modules D (70%) and E (68%) were primary contributors to the conidial germination process (Fig. 4a). This distinctiveness of expression profiles and functional affiliations among different modules indicates that the *F. graminearum* GRN has clear modularity and that each module is differentially expressed under certain biological conditions.

### The *F. graminearum* GRN reveals conserved vs species‐specific components

Novel traits, such as overcoming host resistance to establish infection, or utilizing different nutrient sources to support growth in a changing environment, are important for the adaptation of an organism and, in many cases, are gained through the acquisition of new genes. The integration of these new genes into the regulatory network is crucial for their functionality. Based on a knowledge of the structural and functional divergence of the *F. graminearum* genome (Cuomo *et al*., 2007), we identified 9700 orthologous genes that were conserved among three Fusarium sister species, *F. graminearum*,* F. verticillioides* and *F. oxysporum* (Ma *et al*., 2010), as core genes. About 3600 genes that lack orthologous sequences in the sister species were loosely defined as *F. graminearum* specific (FS hereafter). These FS genes were primarily localized to genomic regions that contained a high density of single nucleotide polymorphisms (SNPs), and some of these genes were found to encode pathogenicity‐related effector proteins (Kistler *et al*., 2013) and secondary metabolite gene clusters (Zhao *et al*., 2014).

Based on this genome partition, we examined whether the regulatory modules identified in the *F. graminearum* GRN had an enrichment of genes located on either genomic region. Interestingly, FS genes are significantly enriched in the three pathogenesis‐related modules, B (30%, *P *= 1.1e‐3), C (52%, *P *= 1.3e‐71) and H (38.2%, *P *= 1.2e‐17), but under‐represented in all other modules (Table 2). Such a biased distribution is reflected in both regulators (Fig. 4b) and their target genes, and the most significant enrichments are observed among FS target genes that are regulated by FS regulators. For instance, 55% of FS target genes in module B, 84% of FS target genes in module C and 73% of FS target genes in module H are regulated by FS regulators.

**Figure 5:**
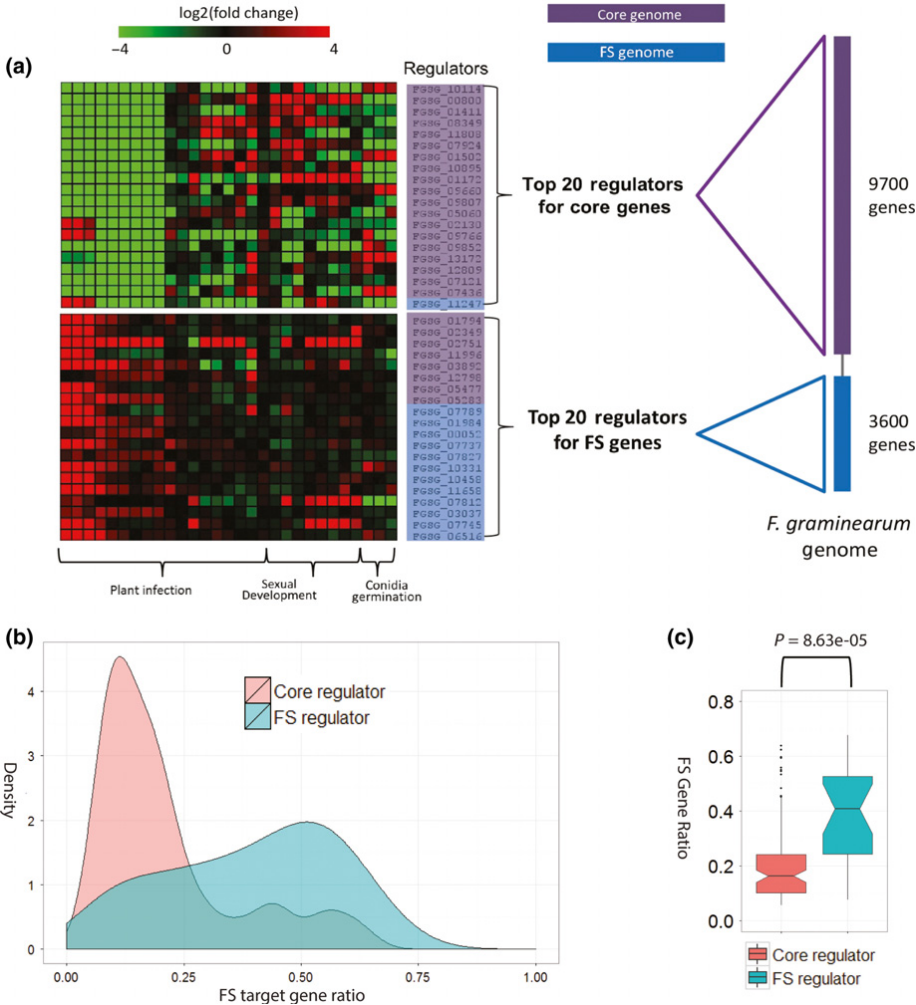
The *Fusarium graminearum* gene regulatory network (GRN) has core genome‐ and *F. graminearum*‐specific (FS) components. (a) Top 20 regulators (in terms of having the most target genes) of target genes belonging to the core genome and FS genome. Purple and blue rectangles denote the core genome and FS genome, respectively. The same colors were also used to shade the top 20 regulators, based on their location in genome compartments. Heatmaps show the gene expression changes of the 40 regulators across three categories of biological conditions. Color scale represents log_2_‐transformed fold change from low (−4) to high (4). Green, downregulated; red, upregulated; black, no change. The core and FS genomes were defined based on a comparative genome analysis between *F. graminearum* and three additional Fusarium species: *F. oxysporum* f. sp. *lycopercisi*,* F. verticillioides* and *F. solani*. Core genes are shared by the four species. FS genes are unique to *F. graminearum*. (b) Kernel density curve of FS target gene ratio distributions for 96 core and 24 FS regulators in the *F. graminearum* GRN. FS target gene ratios measure the proportion of FS genes amongst all target genes per regulator. (c) Notch boxplots summarizing FS target gene ratio distributions for 96 core and 24 FS regulators, which are significantly different, with *P* < 0.05 (Student's *t*‐test). A horizontal black line across each box denotes the mean gene ratio.

To further understand the compartmentalization of regulation, we ranked the top regulators based on their number of target genes, and searched for the top 20 regulators for the conserved vs species‐specific genes, respectively. For the 9700 core genes, 19 of the 20 top regulators were also encoded in the core regions, indicating significant enrichment if assuming a random distribution (*P *= 0.02281). By contrast, only eight of the highest ranked 20 regulators for the 3600 FS genes were found to be core regulators, and 12 were FS regulators (Fig. 5a), which were significantly enriched (*P* = 0.002) (see the Materials and Methods section). This over‐representation of core and FS regulators for the core and FS genomes, respectively, suggests that each genomic region has a regulatory network that primarily employs regulators from the same region. In addition, gene expression patterns for the top 20 regulators of the two genomic regions were also distinct. In contrast with the top regulators in the core genome, those in the FS genome were highly upregulated during plant infection stages, further suggesting that the FS genome and its top regulators play vital roles in fungal pathogenesis (Fig. 5a).

Furthermore, we found that if a regulator was *F. graminearum* specific, its target genes were mostly *F. graminearum* specific, and vice versa. We calculated the core and FS target gene ratios, which represent the frequency of target genes from the core or FS genome, respectively, in the total number of target genes per regulator. Density curves suggested that 96 core regulators (regulators from the core genome) had low FS target gene ratios, compared with 24 FS regulators (Fig. 5b). The FS gene ratio distributions for 24 FS regulators (mean, 0.41) and 96 core regulators (mean, 0.18) were significantly different (Student's *t*‐test: *P *= 8.63e‐05) (Fig. 5c). By contrast, target genes inferred for core regulators were mostly situated in the core genome, and the FS regulators had low core target gene ratios (Fig. S7). These findings indicate that a compartmentalization of regulatory networks exists between the core and FS genomic regions.

### A comparison of the *F. graminearum* GRN and FPPI reveals subnetworks

Many regulatory interactions are effectively carried out by protein complexes, which are essential molecular machineries for a vast array of biological functions. FPPI, which interconnects 3750 proteins, was established previously (Zhao *et al*., 2009). By comparing the *F. graminearum* GRN with the reported FPPI, we identified shared components that capture a total of 311 interactions between 337 proteins. Although this shared network contained only 337 proteins, 78 were predicted top regulators, including 34 SP regulators and 44 TF regulators identified in this study (Fig. S8; Table S8).

These shared interactions formed several major subnetworks, and the top two were a ribosomal protein complex and a complex that involved a subunit of CK2 (FGSG_00677). The ribosomal protein complex contained 30 proteins that were all annotated as ribosomal protein subunits and predicted to be part of module D, which was found to be involved in translation. The subnetwork centering on the regulator FGSG_00677 included a total of 20 proteins and was part of module E, which was found to control transcription and cell transport. FGSG_00677, an essential *F. graminearum* kinase (Wang *et al*., 2011), was found to be involved in cell cycle control, rRNA synthesis, and cell growth and polarity (Table S4). Overall, we found that proteins involved in the cell cycle and transcription were enriched in this subnetwork. Consistent with the active involvement of protein synthesis, DNA synthesis and transcription during fungal spore germination (Osherov & May, 2001; Seong *et al*., 2008), both modules D and E were strongly transcriptionally associated with conidial germination (Fig. 4a).

The other shared component had a key regulator, adenylate cyclase *FAC1* (FGSG_01234), which controls the central signaling cascade cyclic adenosine monophosphate–protein kinase A (cAMP–PKA) pathway by producing the second messenger cAMP (D'Souza & Heitman, 2001) to transmit extracellular stimuli and govern cell responses (Hu *et al*., 2014; Guo *et al*., 2016). This component was part of module A in the *F. graminearum* GRN and was predicted to regulate the cell cycle and cell development.

**Figure 6:**
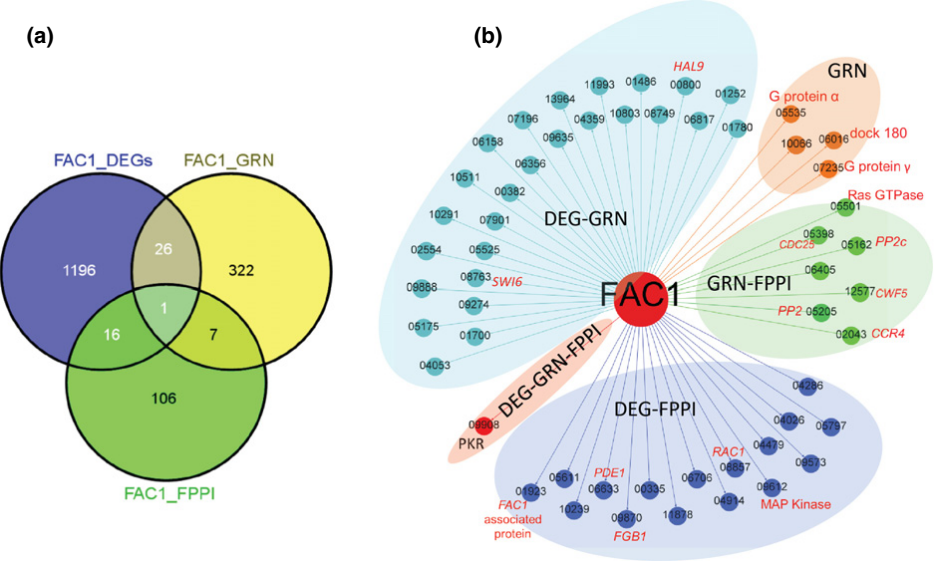
*Fusarium graminearum FAC1* regulatory subnetworks. (a) Venn diagram of network edges captured by the gene regulatory network (GRN), *F. graminearum* protein–protein interaction network (FPPI) and differentially expressed genes (DEG) for the *∆fac1* mutant. (b) *Fusarium graminearum FAC1* (FGSG_01234, adenylate cyclase) regulatory subnetworks. Edges are nonweighted and depict regulatory relationships. For simplicity, prefixes (FGSG_) of genes are omitted. Five clusters within the network are: GRN shared with DEG (DEG‐GRN), GRN shared with FPPI (GRN‐FPPI), FPPI shared with DEG (DEG‐FPPI), shared by all three (DEG‐GRN‐FPPI) and only in GRN (all are not shown). Colors of nodes (except *FAC1*, the regulator) are the same as the clusters. Red labels beside the nodes are either gene names or functional annotations of genes.

Previously, our comparative study of the cAMP–PKA pathway in *F. graminearum* and in one of its sister species, *F. verticillioides* (Guo *et al*., 2016), has revealed that this pathway has conserved housekeeping functions, such as cell cycle regulation, primary metabolism and stress response. In the *F. graminearum* GRN, *FAC1* was predicted to be one of the top regulators, and was found to regulate 356 target genes that were enriched for functions in small GTPase‐mediated signaling, cell cycle regulation and ascospore development (Table S4). Combining FPPI, our GRN inference and the 1238 differentially expressed genes (DEGs) identified in the *Δfac1* mutant (Guo *et al*., 2016), we identified a single edge that was supported by all three lines of evidence (DEG–GRN–FPPI) (Fig. 6a,b). This single node (FGSG_09908) encoded the regulatory subunit of PK (PKR), and binding of cAMP to this subunit led to activation of *CPK1*. Most edges were supported by both GRN and DEG (26 edges), including FGSG_00800 (HAL9) and FGSG_08763 (SWI6). Seven edges were supported by GRN and FPPI, including a RAS GTPase (FGSG_05501) and protein phosphatases PP2, PP2c and CDC25. Interestingly, 16 edges were supported by DEG and FPPI, but not by GRN prediction, including a FAC1‐associated protein (FGSG_01923), the cAMP scavenger enzyme PDE1 (FGSG_06633), G‐protein β subunit FGB1 (FGSG_09870) and a MAPK (FGSG_09612). This suggests that these proteins may be part of a large protein complex involved in the cAMP–PKA signaling pathway, but may not be directly regulated by *FAC1* (Fig. 6b).

Because this current model links all target genes with a subset of regulators, we anticipate a relatively high false‐positive rate in target prediction. Therefore, it is not surprising that we identified over 300 targets that only existed in GRN and lacked support from either DEG or FPPI. These target genes had multiple regulators in GRN; therefore, it is likely that their expression levels may not be changed by blocking a single regulator. However, the GRN‐unique edges did capture cAMP signaling‐related genes, such as the G‐protein α subunit (FGSG_05535) and γ subunit (FGSG_07235), consistent with the fact that G‐protein signaling regulates cAMP signaling. *FAC1* is likely to be co‐regulated with these target genes.

Overall, the network analysis reported here captures many edges that can be supported by both protein–protein interaction and gene expression profiles. Combining these multiple layers of evidence helps us to understand the hierarchical structure of the *FAC1* regulatory network in depth. This analysis provides a template for functional validation of GRN, which could be achieved using genetic and physical interaction data obtained in future biological experiments.

## Discussion

The reconstruction of a GRN at the genomic level remains a daunting task in any organism, particularly in higher eukaryotes. In this study, we selected a filamentous fungus, *F. graminearum*, for the network inference, because of its agricultural importance, the wealth of available transcriptomic data and the existence of a well‐annotated genome. Furthermore, *F. graminearum* is one of the most extensively researched plant‐pathogenic fungi (Dean *et al*., 2012). This first reconstructed GRN of a filamentous ascomycete identified 120 top regulators that regulate different sets of target genes, and provided a regulatory framework that links every gene in the genome. Phenotypic association analysis showed that some of the identified top regulators are critical for fungal asexual and sexual growth, toxin production and virulence. Interestingly, although the SP regulators investigated to date seem to be crucial or indispensable, most TF regulator mutants have similar phenotypes to the wild‐type, with a few exceptions. This may be attributed to different modes of action for these two different types of regulator. SPs, such as kinases, typically act upstream of TFs and are therefore higher on the hierarchy of regulators, behaving as molecular switches. However, TFs often require the cooperation of multiple transcriptional cofactors, as this perhaps allows cells to cope with the loss of certain individual TFs. Regardless, the importance of kinases vs TFs is not always intuitive and, in many cases, requires further investigation.

One significant discovery of this relatively preliminary network inference is the modularity of the network. Any biological function is accomplished by hundreds of proteins through independent, but interconnected, subnetworks. Even though complete separation may be impossible, certain subnetworks are more interconnected than others. Our modularity analysis reproducibly identified eight regulatory modules. Although this distinction between modules is clear, it is not uncommon to find genes associated with more than one module, reflecting the complexity of regulatory networks. The study of these modules will provide valuable insight into the gene regulatory circuits that control key fungal biological processes, and guide the functional characterization of these regulators and target genes through experimental approaches.

The other intriguing discovery of this study is the compartmentalization of the regulatory network. Previous studies have divided the *F. graminearum* genome into conserved vs highly variable genomic regions. Genes encoded within these regions are enriched for species‐specific genes and many of these encode potential virulence factors that are induced during plant infection (Cuomo *et al*., 2007; Zhao *et al*., 2014). In agreement with the genomic division, these two regions are controlled by compartmentalized regulatory circuits in the predicted *F. graminearum* GRN. However, there are strong connections between these two regions and they clearly act as one unit. How *F. graminearum* acquired these novel regulatory circuits during evolutionary processes, how they were integrated with core circuits to form a cohesive functional unit and, finally, how the two parts communicate to regulate diverse fungal biological processes remain to be determined.

Our network inference also provides insight into the TF binding sites for the predicted TF regulators using a purely computational approach. Based on previously published results of an *in silico cis*‐element prediction for *F. graminearum* (Kumar *et al*., 2010) and on homology with *S. cerevisiae*, we found strong conservation signals for 12 TFs (of 75 predicted top TF regulators) and their corresponding TFBSs (matching score > 0.8) between *F. graminearum* and *S. cerevisiae*, indicating functional conservation over > 400 million yr of evolutionary time. As expected, we observed sequence divergence for the binding sites, even when the TFs were conserved, as TF binding site turnover is commonly observed during fungal species evolution, even between closely related species (Gasch *et al*., 2004).

Overall, this network prediction illustrates the power of computational biology and demonstrates the integration of social network and life sciences. However, this current static network model has its limitations. First, it does not consider the dynamic nature of the network and lacks the fine resolution needed to identify regulatory relationships under specific conditions. For example, two well‐characterized TFs, *Tri6* and *Tri10*, which control the production of the mycotoxin trichothecene, are absent in the top regulator list. This is probably a result of their specialized, but nonessential, regulation of a set of genes that control secondary metabolism. Indeed, when we extended our search, both *Tri6* and *Tri10* were identified among the top 500 regulators. Second, the current algorithm is based on the consistent correlation in expression levels of a regulator and target genes without any consideration of post‐transcriptional and post‐translational regulation events, which may have created noise in the prediction. Third, certain genes that share similar patterns of expression without actual regulatory relationships may have been assigned regulatory relationships as false positives. As expression‐based modeling identifies regulators that are active (up‐ or downregulated) under most, if not all conditions, candidate regulators lacking significant expression changes are unlikely to be identified. Despite these limitations, we are confident of the overall accuracy of this network model, which has already revealed exciting and biologically meaningful insights into the gene regulation of this filamentous fungus. By increasing the number of datasets obtained under more diverse conditions, the network structure and topology can be further refined with improved resolution. The identification of the components and dynamics of the cell regulatory network and the pinpointing of specific regulators that govern fungal pathogenesis will offer potential novel strategies to control FHB, one of the most devastating diseases of wheat.

## Author contributions

The project was designed by L. Guo, G.Z., L. Gao and L‐J.M. The manuscript was written by L. Guo, G.Z., L. Gao and L‐J.M. The experimental design was conducted by L. Guo, G.Z., H.C.K., J‐R.X. and L‐J.M. Chip design was conducted by H.C.K., J‐R.X. and L‐J.M.

## Supporting information

Please note: Wiley Blackwell are not responsible for the content or functionality of any supporting information supplied by the authors. Any queries (other than missing material) should be directed to the *New Phytologist* Central Office.

**Fig. S1** Scatterplot matrix of three biological replicates and associated Pearson correlation coefficients (*r*) for each condition/biological state transcriptomically profiled in this study using the Affymetrix Fungal Multigenome ExonChip.
**Fig. S2** Bayesian network score distribution for the top 300 regulators in the *Fusarium graminearum* gene regulatory network (GRN).
**Fig. S3** Visualization of the *Fusarium graminearum* gene regulatory network (GRN) featuring the top 120 regulators, including transcription factors (TFs) and signal proteins (SPs), represented by red and green nodes, respectively.
**Fig. S4** Gene–phenotype networks depicting the association of known phenotypes (red nodes) with inferred signal protein (SP) (blue nodes) and transcription factor (TF) (green nodes) regulators.
**Fig. S5** Regulatory modules are differentially regulated in response to various biological conditions.
**Fig. S6** Heatmap of hierarchical co‐clustering of biological conditions (rows) and 120 regulators (columns).
**Fig. S7** Biased regulatory networks for core regulators of *Fusarium graminearum*.
**Fig. S8** Shared networks by the *Fusarium graminearum* gene regulatory network (GRN) and protein–protein interaction network (FPPI).
**Table S1** Summary of conditions on which the transcriptomic data are based for *Fusarium graminearum* gene regulatory network inference in this study
**Table S4** Summary of signal proteins identified as inferred regulators in this network

**Table S2** Input gene expression data matrix
**Table S5** List of 75 inferred transcription factor regulators and the most enriched binding sites for target genes
**Table S8** Shared networks by the *Fusarium graminearum* gene regulatory network (GRN) and protein–protein interaction network (FPPI)

**Table S3** Spreadsheets containing lists of candidate regulators, inferred 120 top regulators, the full gene regulatory network (GRN), core genome GRN and *Fusarium graminearum*‐specific GRN

**Table S6** Gene lists of eight regulatory modules (A–H) and complete FunCat analysis results

**Table S7** Gene regulatory networks for candidate effector genes
