# Supplementary material for: Compartmentalized gene regulatory network of the pathogenic fungus Fusarium graminearum
Source: New Phytol. 2016 Mar 14;211(2):527–41. doi: 10.1111/nph.13912 (PMC5069591; doi:10.1111/nph.13912)
Supplement: Supplementary file 1 — Fig. S1 Scatterplot matrix of three biological replicates and associated Pearson correlation coefficients (r) for each condition/biological state transcriptomically profiled in this study using the Affymetrix Fungal Multigenome ExonChip. Fig. S2 Bayesian network score distribution for the top 300 regulators in the Fusarium graminearum gene regulatory network (GRN). Fig. S3 Visualization of the Fusarium graminearum gene regulatory network (GRN) featuring the top 120 regulators, including transcription factors (TFs) and signal proteins (SPs), represented by red and green nodes, respectively. Fig. S4 Gene–phenotype networks depicting the association of known phenotypes (red nodes) with inferred signal protein (SP) (blue nodes) and transcription factor (TF) (green nodes) regulators. Fig. S5 Regulatory modules are differentially regulated in response to various biological conditions. Fig. S6 Heatmap of hierarchical co‐clustering of biological conditions (rows) and 120 regulators (columns). Fig. S7 Biased regulatory networks for core regulators of Fusarium graminearum. Fig. S8 Shared networks by the Fusarium graminearum gene regulatory network (GRN) and protein–protein interaction network (FPPI). Table S1 Summary of conditions on which the transcriptomic data are based for Fusarium graminearum gene regulatory network inference in this study Table S4 Summary of signal proteins identified as inferred regulators in this network [file NPH-211-527-s001.pdf]

New Phytologist

New Phytologist Supporting Information Figs S1–S8 and Tables S1–S8

Article title: Compartmentalized gene regulatory network of the pathogenic fungus *Fusarium graminearum*

Authors: Li Guo, Guoyi Zhao, Jin-Rong Xu, H. Corby Kistler, Lixin Gao and Li-Jun Ma

Article acceptance date: 25 January 2016

The following Supporting Information is available for this article:

Fig. S1 Scatterplot matrix of three biological replicates and associated Pearson correlation coefficients (*r*) for each condition/biological state transcriptomically profiled in this study using the Affymetrix Fungal Multigenome ExonChip.

Fig. S2 Bayesian network score distribution for the top 300 regulators in the *Fusarium graminearum* GRN.

Fig. S3 Visualization of the *Fusarium graminearum* GRN featuring the top 120 regulators, including TFs and SPs, represented by red and green nodes, respectively.

Fig. S4 Gene–phenotype networks depicting the association of known phenotypes (red nodes) with inferred SP (blue nodes) and TF regulators (green nodes).

Fig. S5 Regulatory modules are differentially regulated in response to various biological conditions.

Fig. S6 Heatmap of hierarchical co-clustering of biological conditions (rows) and 120 regulators (columns).

Fig. S7 Biased regulatory networks for core regulators of *Fusarium graminearum*.

Fig. S8 Shared networks by the *Fusairum graminearum* GRN and protein–protein interaction network (FPPI).

Table S1 Summary of conditions on which the transcriptomic data are based for *Fusarium graminearum* gene regulatory network inference in this study

Table S2 Input gene expression data matrix (separate Excel file)

Table S3 Spreadsheets containing lists of candidate regulators, inferred 120 top regulators, the full GRN, core genome GRN, and *Fusarium graminearum*-specific GRN (separate Excel file)

Table S4 Summary of signaling proteins identified as inferred regulators in this network

Table S5 List of 75 inferred transcription factor regulators and the most enriched binding site for target genes (separate Excel file)

Table S6 Gene lists of eight regulatory modules (A–H) and complete FunCat analysis results (separate Excel file)

Table S7 Gene regulatory networks for candidate effector genes (separate Excel file)

Table S8 *Fusarium graminearum* GRN and FPPI shared networks (separate Excel file)

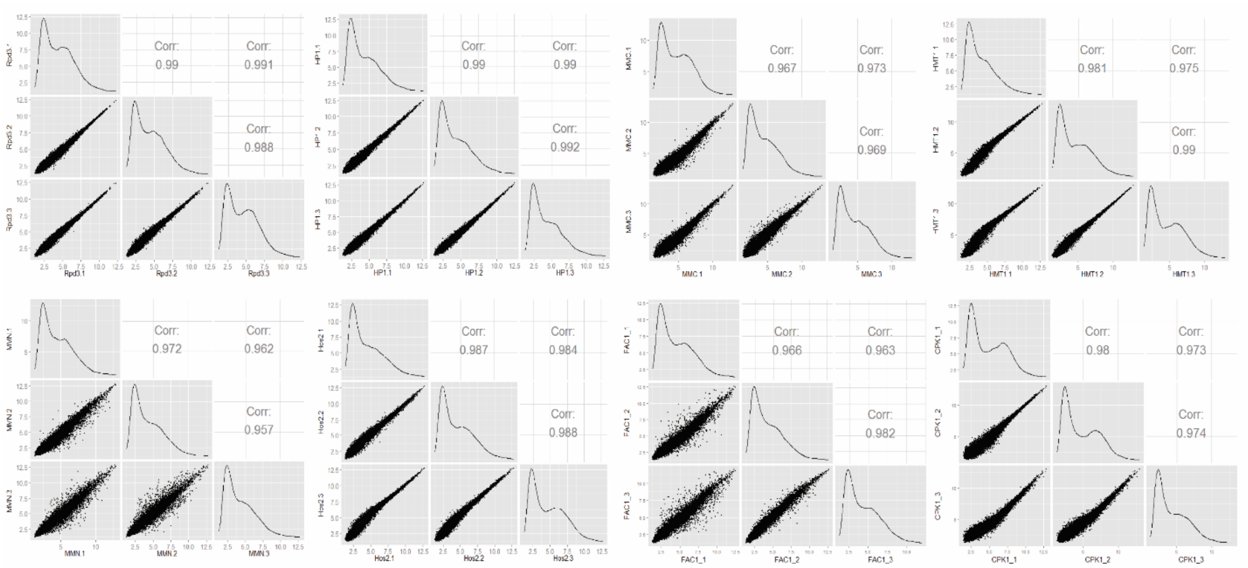

Fig. S1 Scatterplot matrix of three biological replicates and associated Pearson correlation coefficients (*r*) for each condition/biological state transcriptomically profiled in this study using the Affymetrix Fungal Multigenome ExonChip. Conditions/biological states include: complete medium (CM), carbon starvation medium (MMC), nitrogen starvation medium (MMN), and the *HMT1*, *Hos2*, *Rpd3*, *HP1*, *CPK1,* and *FAC1* mutants. The *x*- and *y*-axis in each scatterplot represent expression values (log_2-transformed). The scatterplot matrix (eight blocks) was created using the R package *GGally*. The Pearson correlation coefficient (*r*) is listed for each scatter plot.

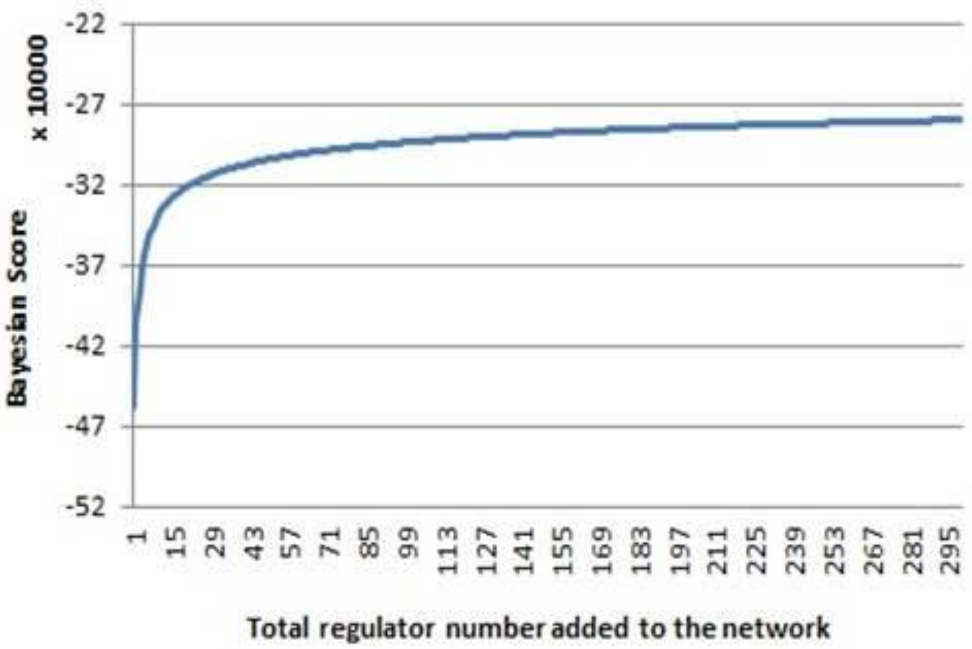

Fig. S2 Bayesian network score (BNS) distribution for the top 300 regulators in the *Fusarium graminearum* gene regulatory network (GRN). *X*- and *y*-axis represent the accumulating number of inferred regulators and the corresponding BNS of the GRN.

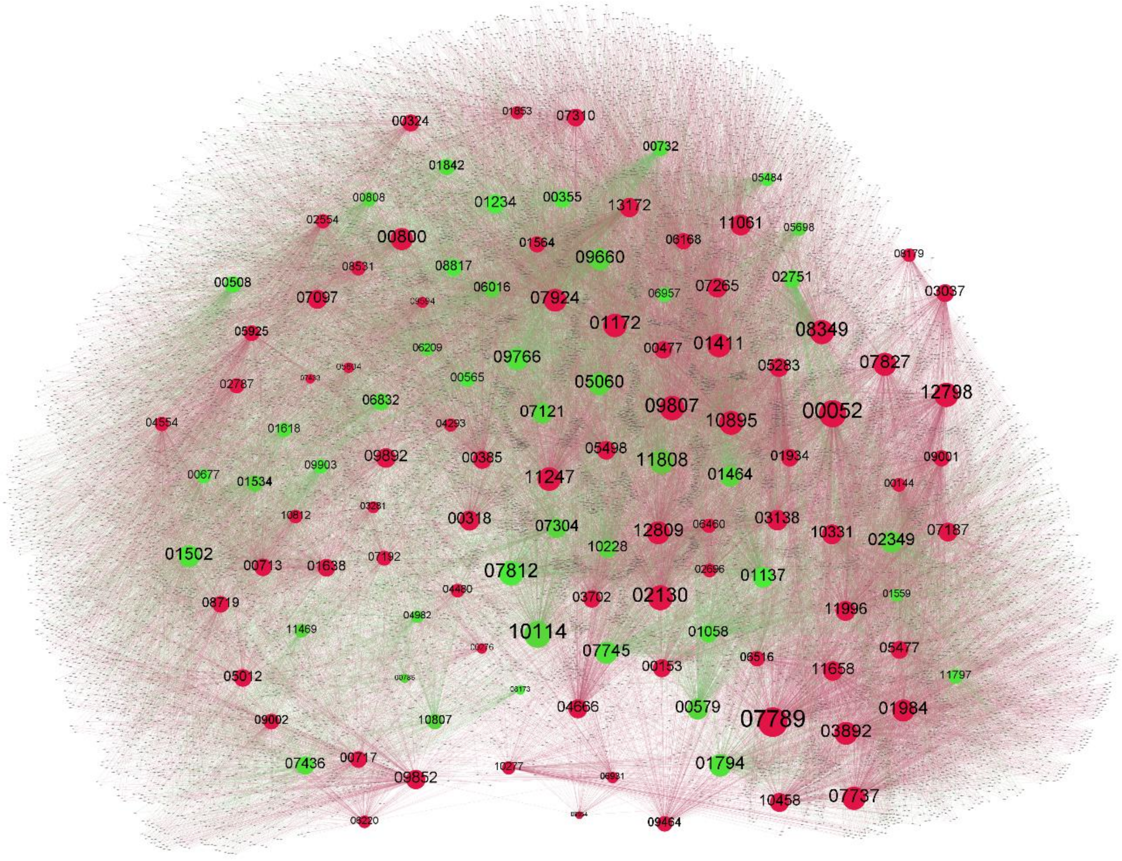

Fig. S3 Visualization of the *Fusarium graminearum* GRN featuring the top 120 regulators, including transcription factors (TF) and signaling proteins (SP), represented by red and green nodes, respectively. Target genes are represented by grey nodes. For simplicity, prefixes (FGSG_) of gene_ID were omitted. Sizes of nodes are proportional to their out-degrees (target gene numbers).

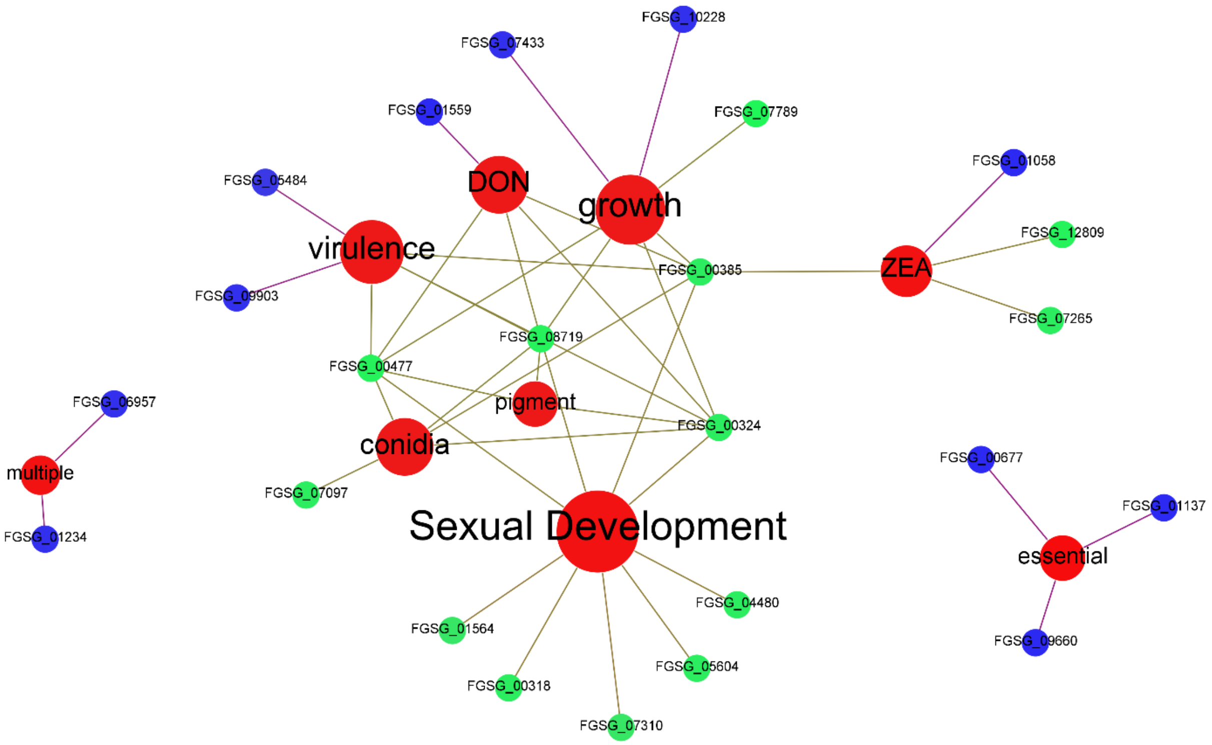

Fig. S4 Gene–phenotype networks depicting the association of known phenotypes (red nodes) with inferred SP (blue nodes) and TF regulators (green nodes). Phenotype information is derived from FgTFPD (Son *et al.*, 2011), a previous kinome analysis (Wang *et al.*, 2011), and the PHI-base (Winnenburg *et al.*, 2008). Phenotypes include conidia (conidia germination), growth (vegetative growth on PDA or minimal media), sexual development (perithecium and ascospore development), deoxynivalenol production (DON), zearalenone production (ZEA), virulence (wheat head blight), multiple (pleiotropic), and essential (lethal mutation).

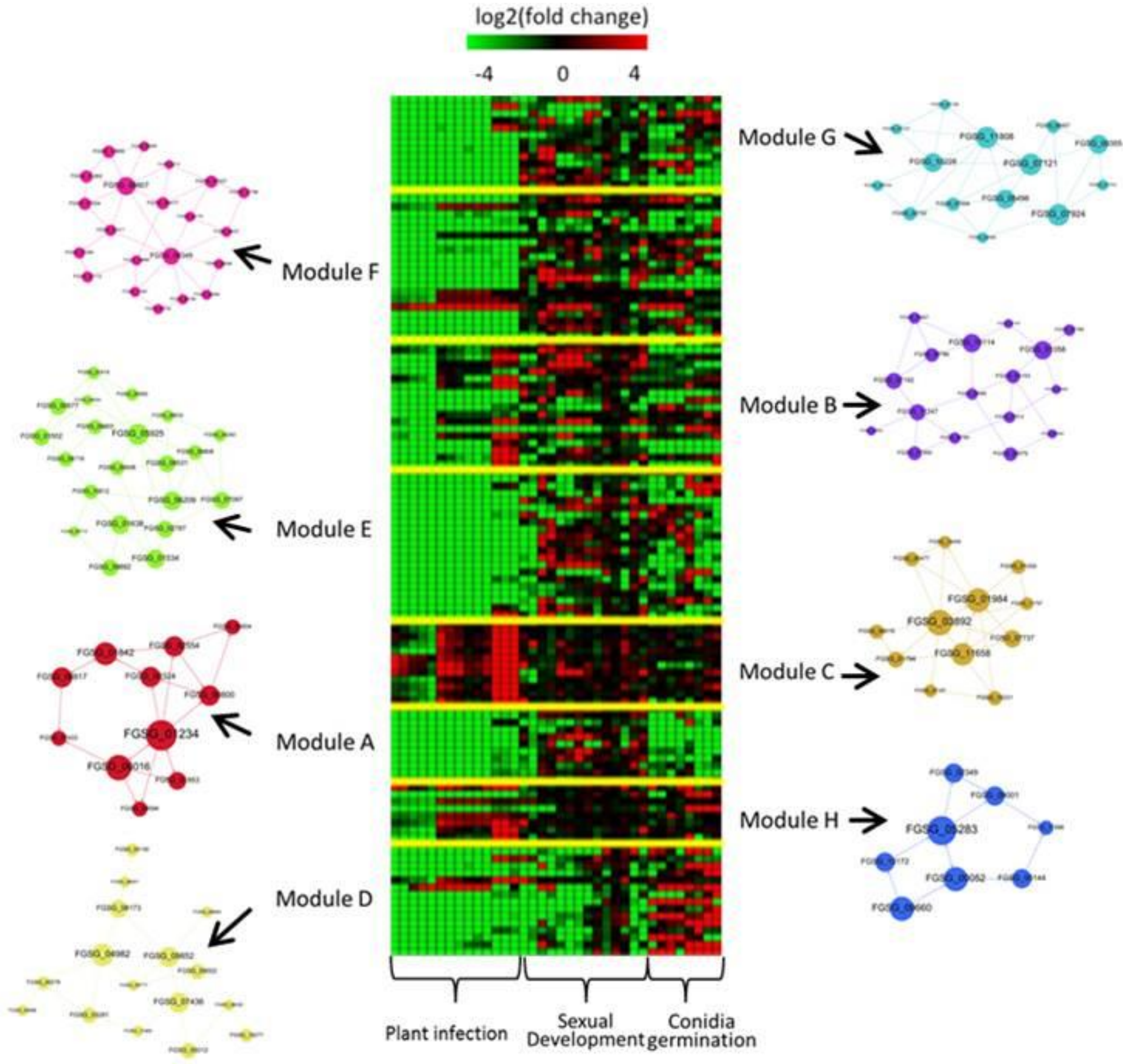

Fig. S5 Regulatory modules are differentially regulated in response to various biological conditions. Gene expression of 120 regulators (rows) was clustered with hierarchical clustering and visualized in a heatmap. Color scale represents log_2-transformed fold change from low (-4) to high (4). Green, downregulated; red, upregulated; black, no change. Biological conditions (columns) are divided into three categories: plant infection, sexual development, and conidia germination. Yellow lines in the heatmap are boundaries for different modules of regulators. Module A–H along with their respective subnetworks (pointed in arrows, extracted from Fig. 3) annotate each section of the heatmap. Color rules and node sizes are the same as in Fig. 3.

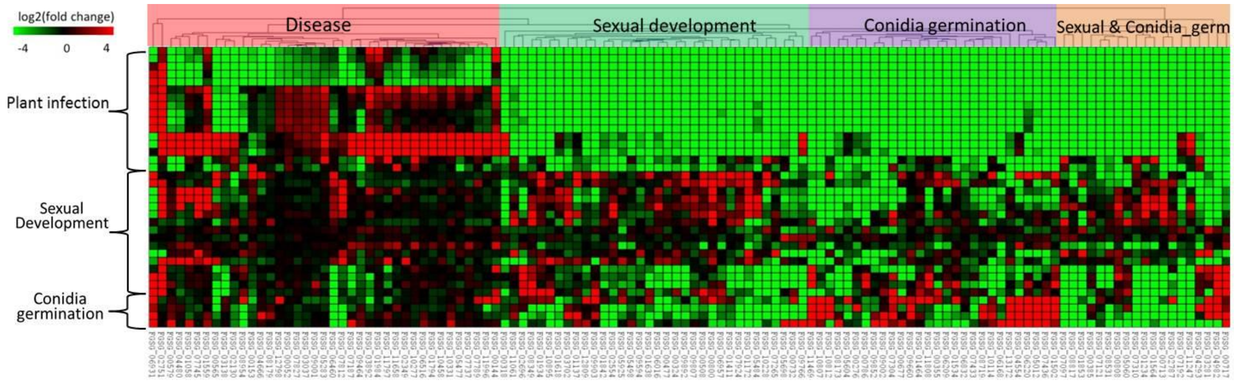

Fig. S6 Heatmap of hierarchical co-clustering of biological conditions (rows) and 120 regulators (columns). Color scale represents log_2-transformed fold change from low (-4) to high (4). Green, downregulated; red, upregulated; black, no change. Biological conditions are clustered into three categories: plant infection, sexual development, and conidia germination. Based on up- and downregulation of regulators in different biological condition categories, regulators are clustered into four major clusters: disease, sexual development, conidia germination, and sexual development and conidia germination.

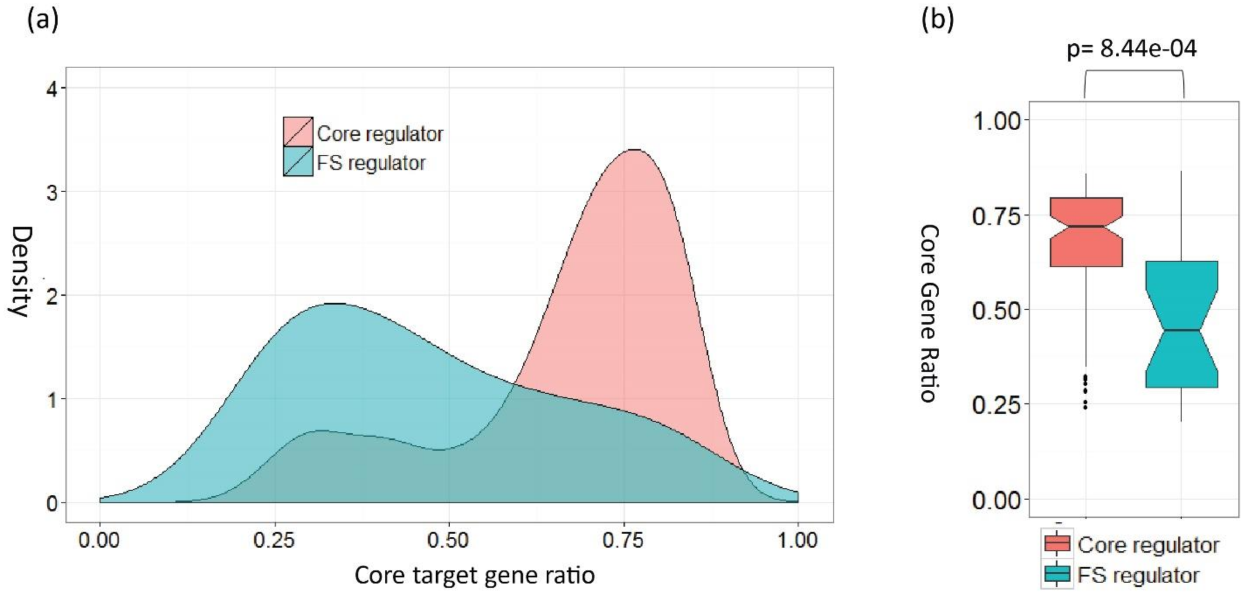

Fig. S7 Biased regulatory networks for core regulators of *Fusarium graminearum*. (a) Kernel density curve of core target gene ratio distributions for 96 core and 24 *F. graminearum*-specific (FS) regulators in the *F. graminearum* GRN. Core target genes ratios measure the proportion of core genes amongst total target genes per regulator. (b) Notch boxplots summarizing core target gene ratio distributions for 96 core and 24 FS regulators, which are significantly different with a *P*-value of < 0.05 (Student's *t*-test). The horizontal black line across each box denotes the mean gene ratio.

New Phytologist

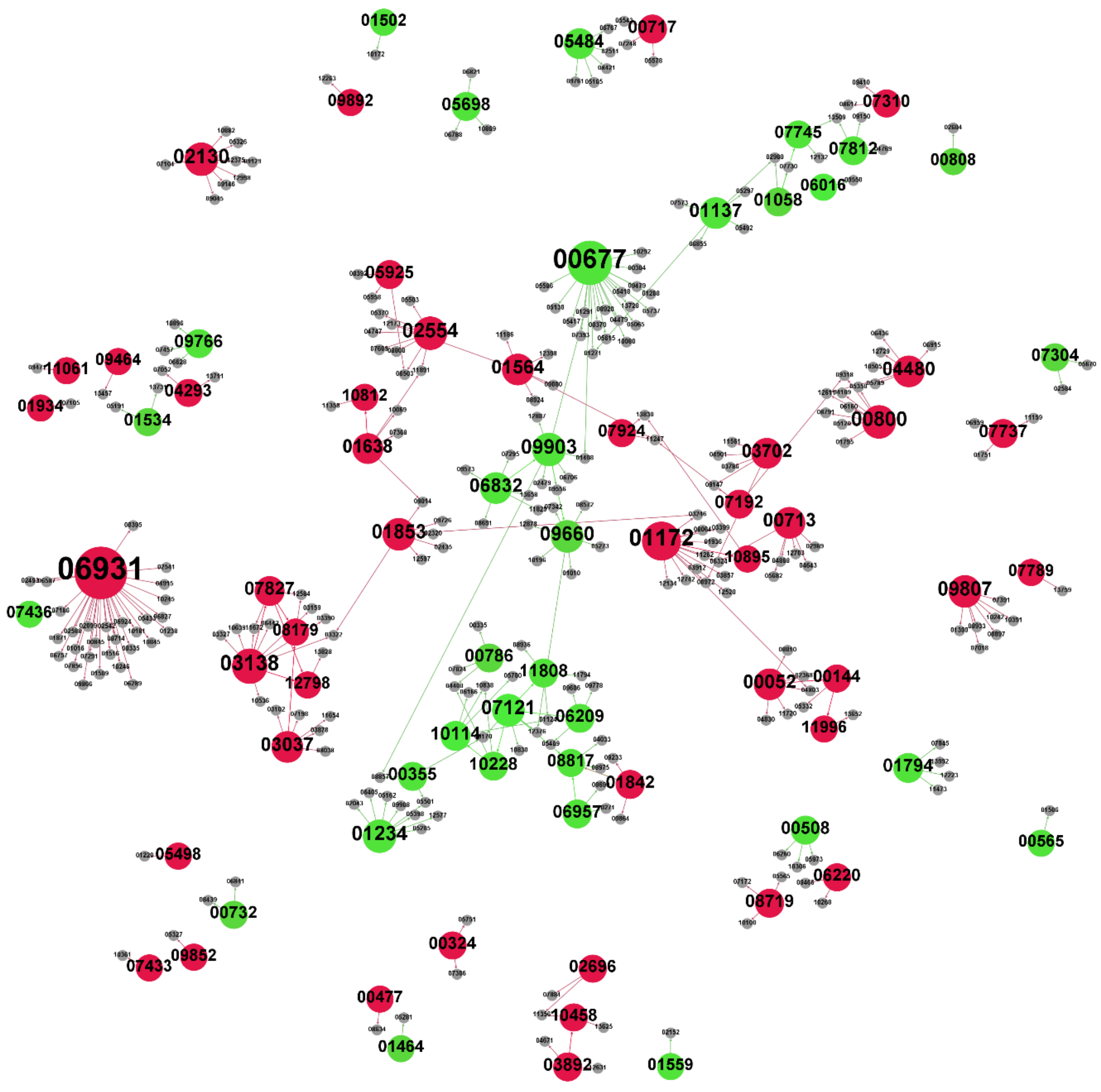

Fig. S8 Shared networks by the *Fusairum graminearum* GRN and protein–protein interaction network (FPPI). For simplicity, prefixes (FGSG_) of gene_ID were omitted. Size of nodes are proportional to their out-degrees (target gene numbers). Red nodes, transcription factor regulators; green nodes, signaling protein regulators; grey nodes, interacting proteins/target genes.

New Phytologist

Table S1 Summary of conditions on which the transcriptomic data are based for *Fusarium graminearum* GRN inference in this study

Experiment	Condition ID	Biological conditions	No. of biological replicates	Source
Nutrient starvation	CM*	Complete medium (CM)	3	This study
Nutrient starvation	MMC	Carbon starvation	3	This study
Nutrient starvation	MMN	Nitrogen starvation	3	This study
Gene perturbation	FAC1	Adenylyl cyclase mutant growing on CM	3	This study
Gene perturbation	CPK1	Protein kinase A mutant growing on CM	3	This study
Gene perturbation	Hos2	*Hos2* mutant growing on CM	3	This study
Gene perturbation	HMT1	*HMT1* mutant growing on CM	3	This study
Gene perturbation	Rpd3	*Rpd3* mutant growing on CM	3	This study
Gene perturbation	HP1	*HP1* mutant growing on CM	3	This study
FG18	FG18_H4-H6	Fgp1 mutant on Putrescine	3	PLEXdb
FG18	FG18_H1-H3*	Wild type on Putrescine	3	PLEXdb
FG16	FG16_H1-H3*	Vegetative hyphae	3	PLEXdb
FG16	FG16_H4-H6	Wide dikaryotic hyphae	3	PLEXdb
FG16	FG16_H7-H9	Perithecial initials	3	PLEXdb
FG16	FG16_H10-H12	Young perithecia	3	PLEXdb
FG15	FG15_H1-H3*	Wheat control	3	PLEXdb
FG15	FG15_H4-H6	24 h infection	3	PLEXdb
FG15	FG15_H7-H9	48 h infection	3	PLEXdb
FG15	FG15_H10-H12	72 h infection	3	PLEXdb
FG15	FG15_H13-H15	96 h infection	3	PLEXdb
FG15	FG15_H16-H18	144 h infection	3	PLEXdb
FG15	FG15_H19-H21	192 h infection	3	PLEXdb
FG14	FG14_H1-H4	Agmatine	4	PLEXdb

FG14	FG14_H5-H8*	Glutamine	4	PLEXdb
FG13	FG13_H1-H3*	WT PH1 CMC media	3	PLEXdb
FG13	FG13_H4-H6	WT PH1 wheat infection	3	PLEXdb
FG13	FG13_H7-H9	WT PH1 Secondary metabolism	3	PLEXdb
FG13	FG13_H10-H12	ΔFgStuA CMC media	3	PLEXdb
FG13	FG13_H13-H15	ΔFgStuA wheat infection	3	PLEXdb
FG13	FG13_H16-H18	ΔFgStuA Secondary mtabolism	3	PLEXdb
FG12	FG12_H1-H4	Crown rot infection 2 dpi	4	PLEXdb
FG12	FG12_H5-H8	Crown rot infection 14 dpi	4	PLEXdb
FG12	FG12_H9-H11	Crown rot infection 35 dpi	3	PLEXdb
FG12	FG12_H12-H15*	Mycelia cultured	4	PLEXdb
FG11	FG11_H1-H3*	Infection PH-1	3	PLEXdb
FG11	FG11_H4-H6	Infection PH-1 Tri6 deletion	3	PLEXdb
FG11	FG11_H7-H9	Infection PH-1 Tri10 deletion	3	PLEXdb
FG10	FG10_H1-H3	Trochodien 250uM	3	PLEXdb
FG10	FG10_H4-H6*	No trochodien	3	PLEXdb
FG7	FG7_H1-H3*	Conidia germination 0 h	3	PLEXdb
FG7	FG7_H4-H6	Conidia germination 2 h	3	PLEXdb
FG7	FG7_H7-H9	Conidia germination 8 h	3	PLEXdb
FG7	FG7_H10-H12	Conidia germination 24 h	3	PLEXdb
FG6	FG6_H1-H3*	Mutant cch1-14 sexual development 0 h	3	PLEXdb
FG6	FG6_H4-H6	Mutant cch1-14 sexual development 96 h	3	PLEXdb
FG6	FG6_H7-H9	Mutant cch1-14 sexual development 144 h	3	PLEXdb
FG5	FG5_H1-H5*	PH-1 sexual development 0 h	5	PLEXdb
FG5	FG5_H6-H8	PH-1 sexual development 24 h	3	PLEXdb
FG5	FG5_H11-H14	PH-1 sexual development 48 h	4	PLEXdb
FG5	FG5_H16-H18	PH-1 sexual development 72 h	3	PLEXdb
FG5	FG5_H21-H25	PH-1 sexual development 96 h	5	PLEXdb
FG5	FG5_H26-H28	PH-1 sexual development 144 h	3	PLEXdb
FG1	FG1_H1-H3*	Barley infection water control	3	PLEXdb
FG1	FG1_H4-H6	Barley infection 24 h	3	PLEXdb
FG1	FG1_H7-H9	Barley infection 48 h	3	PLEXdb
FG1	FG1_H10-H12	Barley infection 72 h	3	PLEXdb
FG1	FG1_H13-H15	Barley infection 96 h	3	PLEXdb
FG1	FG1_H16-H18	Barley infection 144 h	3	PLEXdb
FG2	FG2_H1-H3*	Complete medium	3	PLEXdb
FG2	FG2_H4-H6	Carbon starvation	3	PLEXdb
FG2	FG2_H7-H9	Nitrogen starvation	3	PLEXdb

*Reference condition for each experiment. Note that nutrient starvation and gene perturbation experiments share the same reference condition ‘CM’. dpi, d post infection.

Table S4 Summary of signaling proteins identified as inferred regulators in this network

Regulator	Annotation	Primary GO terms of regulator	Target genes	GRN_FPPI shared	GO terms overlapped	Total regulator GO terms	Total enriched GO terms of target genes	Function capture ratio (R_{GO})
FGSG_00355	RAS GTPase-activating protein SAR1	Cellular signaling small GTPase; mediated signal transduction, Cell growth / morphogenesis; fungal/microorganismic cell type differentiation	321	3	17	24	98	0.7083333333
FGSG_00508	calcium P-type ATPase	Ion transport; electron transport; vesicular transport (Golgi network, etc.); cellular export and secretion; NIK-I-kappaB/NF-kappaB cascade stress response; homeostasis of cations; cell-cell adhesion; actin cytoskeleton	300	3	16	33	114	0.4848484848
FGSG_00565	protein-tyrosine-phosphatase	Phosphate metabolism; cell cycle; G2/M transition of mitotic cell cycle; cytokinesis (cell division); septum formation and hydrolysis protein kinase	264	1	3	25	51	0.12
FGSG_00677	protein kinase ck2 catalytic subunit ck2 alpha-3	Mitotic cell cycle and cell cycle control; rRNA synthesis; modification by ubiquitin-related proteins; cellular signaling; Wnt signaling pathway; DNA damage response; homeostasis; cell-cell adhesion; cell growth / morphogenesis; cytoskeleton/structural proteins; budding, cell polarity and filament formation	242	14	33	48	120	0.6875
FGSG_00732	A.ambisexualis antheridiol steroid receptor	C-compound and carbohydrate metabolism; cellular signaling	288	2	1	4	101	0.25
FGSG_00786	serine/threonine-protein kinase	Phosphate metabolism; respiration; mitotic cell cycle and cell cycle control; meiosis; transcription activation; cellular signaling; Ca^{2+} mediated signal	112	3	1	33	31	0.0303030303

		transduction						
FGSG_00808	GTP-binding protein Rab4b	Purin nucleotide/nucleoside/nucleobase metabolism; phosphate metabolism; protein transport; neurotransmitter transport; vesicular transport (Golgi network, etc.); cellular signaling, G-protein mediated signal transduction; small GTPase mediated signal transduction	257	1	2	19	56	0.1052631579
FGSG_01058	Cell wall biosynthesis kinase	Phosphate metabolism; mitotic cell cycle and cell cycle control; cellular signaling; G-protein coupled receptor signaling pathway; cell growth / morphogenesis; mating (fertilization); cell wall; budding, cell polarity and filament formation	328	3	0	47	24	0
FGSG_01137	protein kinase RPK1	Phosphate metabolism; mitotic cell cycle and cell cycle control; cell cycle checkpoints; meiosis; cytokinesis (cell division)/ septum formation and hydrolysis; spindle pole body/centrosome and microtubule cycle; cellular signaling; cell growth / morphogenesis; cell wall; microtubule cytoskeleton	406	6	6	26	45	0.2307692308
FGSG_01234	adenylate cyclase	Metabolism of cyclic and unusual nucleotides; mitotic cell cycle and cell cycle control; meiosis; protein targeting, sorting and translocation; small GTPase mediated signal transduction; cAMP/cGMP mediated signal transduction; FGF-receptor signalling pathway; perception of nutrients and nutritional adaptation; development of asco- basidio- or zygospore	356	8	5	10	119	0.5
FGSG_01464	cell shape control	Phosphate metabolism; mitotic	398	1	6	31	62	0.1935483871

	protein phosphatase ppe1	cell cycle and cell cycle control; G1/S transition of mitotic cell cycle; cellular signaling; protein kinase; NIK-I-kappaB/NF-kappaB cascade; cell wall; actin cytoskeleton; microtubule cytoskeleton; fungal and other eukaryotic cell type differentiation; budding, cell polarity and filament formation						
FGSG_01502	ACOB protein	Modification by ubiquitin-related proteins; proteasomal degradation (ubiquitin/proteasomal pathway); enzyme mediated signal transduction; development of asco- basidio- or zygospore	430	1	18	22	128	0.8181818182
FGSG_01534	GTP-binding protein rab2	Protein targeting; sorting and translocation; protein transport ER to Golgi transport; vesicle formation cellular signaling; G-protein mediated signal transduction; small GTPase mediated signal transduction; disease, virulence and defense	290	2	11	19	106	0.5789473684
FGSG_01559	CDC5 - protein kinase involved in regulation of DNA replication	Nucleotide/nucleoside/nucleobase metabolism;<br>phosphate metabolism;<br>DNA synthesis and replication;<br>DNA repair;<br>mitotic cell cycle and cell cycle control;<br>cell cycle checkpoints;<br>cellular signaling;<br>apoptosis (type I programmed cell death);<br>cytoskeleton/structural proteins	205	1	0	35	12	0
FGSG_01618	conserved hypothetical protein	Lipid, fatty acid and isoprenoid metabolism;<br>tetracyclic and pentacyclic triterpenes (cholesterin, steroids and hopanoids) metabolism;<br>cellular signaling	255	0	0	6	46	0
FGSG_01794	short-chain	C-compound and carbohydrate	447	4	0	6	20	0

	alcohol dehydrogenase	metabolism;<br>sugar, glucoside, polyol and carboxylate catabolism;<br>pentose-phosphate pathway						
FGSG_01842	protein serine kinases	Phosphate metabolism;<br>regulation of C-compound and carbohydrate metabolism;<br>cellular signaling;<br>MAPKKK cascade;<br>transmembrane receptor protein serine/threonine kinase signalling pathways;<br>apoptosis (type I programmed cell death);<br>microtubule cytoskeleton	286	3	13	30	79	0.4333333333
FGSG_02349	tyrosine-tRNA ligase	Translation;<br>aminoacyl-tRNA-synthetases;<br>interleukine receptor signalling pathway;<br>cell motility;<br>apoptosis (type I programmed cell death)	416	0	0	19	16	0
FGSG_02751	aldehyde reductase	Sugar, glucoside, polyol and carboxylate metabolism;<br>polyphosphoinositol mediated signal transduction;<br>osmosensing and response;<br>eukaryotic plasma membrane	330	0	0	17	16	0
FGSG_04982	protein-tyrosine phosphatase	Phosphate metabolism;<br>mitotic cell cycle and cell cycle control;<br>cellular signaling;<br>polyphosphoinositol mediated signal transduction;<br>cell growth / morphogenesis	195	0	4	16	67	0.25
FGSG_05060	phospholipase C	Lipid, fatty acid and isoprenoid metabolism;<br>phospholipid metabolism;<br>cation transport (H+, Na+, K+, Ca2+ , NH4+, etc.);<br>cellular signaling;<br>second messenger mediated signal transduction;	448 ()	0	3	16	35	0.1875

		fatty acid derivatives mediated signal transduction; polyphosphoinositol mediated signal transduction						
FGSG_05698	WD40-repeat protein (notchless protein)	rRNA processing; ribosome biogenesis; translation; assembly of protein complexes; nuclear transport; Notch-receptor signalling pathway; fungal and other eukaryotic cell type differentiation	233	3	8	23	47	0.347826087
FGSG_06016	dock180 protein	Cellular signaling; small GTPase mediated signal transduction; chemotaxis; eukaryotic plasma membrane; cytoskeleton/structural proteins; actin cytoskeleton	301	1	14	24	86	0.5833333333
FGSG_06209	（SEC4-like Rab/GTPase）	Vesicular transport (Golgi network, etc.); cellular signaling; G-protein mediated signal transduction	228	3	2	7	88	0.2857142857
FGSG_06832	Prk1p, and serine/threonine protein kinase homolog from A. thaliana	Phosphate metabolism; assembly of protein complexes; endocytosis; cellular signaling; serine/threonine kinase; actin cytoskeleton	313	6	21	26	89	0.8076923077
FGSG_07121	serine/threonine-protein kinase Chk2	Phosphate metabolism; DNA repair; cell cycle arrest; cell cycle checkpoints; cellular signaling; protein kinase; Ca2+ mediated signal transduction; DNA damage response; apoptosis (type I programmed cell death)	397	7	4	36	62	0.1111111111
FGSG_07304	conserved	Cell cycle;	379	2	3	12	98	0.25

	hypothetical protein	cellular signaling;<br>JNK cascade;<br>NIK-I-kappaB/NF-kappaB cascade;<br>small GTPase mediated signal transduction						
FGSG_07433	hypothetical protein similar to RhoGAP and Fes/CIP4 domain protein	Signal transduction;<br>small GTPase mediated signal transduction;<br>establishment or maintenance of actin cytoskeleton polarity;<br>intracellular signal transduction	113	1	10	14	112	0.7142857143
FGSG_07436		Translation;<br>aminoacyl-tRNA-synthetases;<br>interleukine receptor signalling pathway;<br>cell motility;<br>apoptosis (type I programmed cell death)	344	1	11	19	130	0.5789473684
FGSG_07812	GDP/GTP exchange factor for Gsp1p/Gsp2p	Cell cycle;<br>Cell division	505	3	1	1	33	1
FGSG_08173	RAS superfamily KREV-1	Mitotic cell cycle and cell cycle control;<br>cell cycle checkpoints;<br>cytokinesis (cell division) /septum formation and hydrolysis;<br>rRNA processing;<br>tRNA processing;<br>mRNA processing (splicing, 5'-, 3'-end processing);<br>ribosome biogenesis;<br>protein modification;<br>modification by ubiquitination, deubiquitination;<br>proteasomal degradation (ubiquitin/proteasomal pathway);<br>protein transport;<br>RNA transport;<br>nuclear transport;<br>cellular signaling	113	0	25	37	77	0.6756756757
FGSG_08817	protein kinase C-like	Purin nucleotide/nucleoside/nucleobase metabolism;	316	2	11	31	100	0.3548387097

		phosphate metabolism;<br>transfer of activated C-1 groups;<br>cell cycle;<br>mitotic cell cycle and cell cycle control;<br>G-protein mediated signal transduction;<br>small GTPase mediated signal transduction;<br>chemotaxis;<br>cell growth / morphogenesis;<br>cytoskeleton/structural proteins;<br>budding, cell polarity and filament formation						
FGSG_09660	putative RNA helicase involved in ribosome biogenesis	C-compound and carbohydrate metabolism;<br>lipid, fatty acid and isoprenoid metabolism;<br>isoprenoid metabolism;<br>metabolism of vitamins, cofactors, and prosthetic groups;<br>glycolysis and gluconeogenesis;<br>electron transport;<br>electromagnetic waves stress response (e.g. UV, X-ray);<br>detoxification by degradation	438	7	22	44	110	0.5
FGSG_09766	MAP kinase kinase	Phosphate metabolism;<br>DNA topology;<br>DNA repair;<br>DNA recombination;<br>DNA conformation modification (e.g. chromatin);<br>RNA synthesis;<br>transcriptional control;<br>transcription repression;<br>RNA processing;<br>rRNA processing;<br>tRNA processing;<br>mRNA processing (splicing, 5'-, 3'-end processing);<br>splicing;<br>control of mRNA stability;<br>ribosome biogenesis;<br>ribosomal proteins;	432	3	5	44	59	0.1136363636

		hormone mediated signal transduction; organization of chromosome structure						
FGSG_09903	MAP kinase kinase	Phosphate metabolism; MAPKKK cascade; serine/threonine kinase; transmembrane signal transduction; perception of nutrients and nutritional adaptation; budding, cell polarity and filament formation	247	8	24	29	146	0.8275862069
FGSG_10114	RAS-2 protein	Purin nucleotide/nucleoside/nucleobase metabolism; regulation of nucleotide/nucleoside/nucleobase metabolism; phosphate metabolism; transfer of activated C-1 groups; regulation of C-compound and carbohydrate metabolism; cell cycle; mitotic cell cycle and cell cycle control; cytokinesis (cell division) /septum formation and hydrolysis; protein kinase; small GTPase mediated signal transduction; cAMP/cGMP mediated signal transduction; stress response; perception of nutrients and nutritional adaptation; directional cell growth (morphogenesis); cell death; cell aging; cell wall; fungal and other eukaryotic cell type differentiation; budding, cell polarity and filament	557	4	24	46	116	0.5217391304

		formation;<br>hyphae formation;<br>development of asco- basidio- or zygospore						
FGSG_10807	NADPH oxidase 1	Electron transport;<br>cellular signaling;<br>superoxide metabolism;<br>homeostasis of cations;<br>gravity perception and response;<br>temperature perception and response	273	0	11	25	79	0.44
FGSG_11469	GTP-binding protein	Cellular signaling	231	0	3	5	107	0.6

References

Son H, Seo YS, Min K, Park AR, Lee J, Jin JM, Lin Y, Cao P, Hong SY, Kim EK *et al*. 2011. A phenome-based functional analysis of transcription factors in the cereal head blight fungus, *Fusarium graminearum*. *PLoS Pathog* **7:** e1002310.

Wang C, Zhang S, Hou R, Zhao Z, Zheng Q, Xu Q, Zheng D, Wang G, Liu H, Gao X *et al*. 2011. Functional analysis of the kinome of the wheat scab fungus *Fusarium graminearum*. *PLoS Pathog* **7:** e1002460.

Winnenburg R, Baldwin TK, Urban M, Rawlings C, Köhler J, Hammond-Kosack KE. 2006. PHI-base: a new database for pathogen host interactions. *Nucleic Acids Research* **34:** D459–D464.
